# Prediction of Site Directed miRNAs as Key Players of Transcriptional Regulators Against Influenza C Virus Infection Through Computational Approaches

**DOI:** 10.3389/fmolb.2022.866072

**Published:** 2022-04-08

**Authors:** Mubashir Hassan, Muhammad Shahzad Iqbal, Sawaira Naqvi, Hany Alashwal, Ahmed A. Moustafa, Andrzej Kloczkowski

**Affiliations:** ^1^ Institute of Molecular Biology and Biotechnology, The University of Lahore, Lahore, Pakistan; ^2^ The Steve and Cindy Rasmussen Institute for Genomic Medicine, Nationwide Children Hospital, Columbus, OH, United States; ^3^ Department of Biotechnology, Faculty of Life Sciences, University of Central Punjab, Lahore, Pakistan; ^4^ College of Information Technology, United Arab Emirates University, Al-Ain, United Arab Emirates; ^5^ Department of Human Anatomy and Physiology, The Faculty of Health Sciences, University of Johannesburg, Johannesburg, South Africa; ^6^ School of Psychology, Faculty of Society and Design, Bond University, Gold Coast, QLD, Australia; ^7^ Department of Pediatrics, The Ohio State University, Columbus, OH, United States

**Keywords:** miRNAs, mirbase, RStudio, RNAComposer, target site prediction, influenza C virus, HEF

## Abstract

MicroRNAs (miRNAs) are small non-coding RNAs that play critical roles in gene expression, cell differentiation, and immunity against viral infections. In this study, we have used the computational tools, RNA22, RNAhybrid, and miRanda, to predict the microRNA-mRNA binding sites to find the putative microRNAs playing role in the host response to influenza C virus infection. This computational research screened the following four miRNAs: hsa-mir-3155a, hsa-mir-6796-5p, hsa-mir-3194-3p and hsa-mir-4673, which were further investigated for binding site prediction to the influenza C genome. Moreover, multiple sites in protein-coding region (HEF, CM2, M1-M2, NP, NS1- NS2, NSF, P3, PB1 and PB2) were predicted by RNA22, RNAhybrid and miRanda. Furthermore, 3D structures of all miRNAs and HEF were predicted and checked for their binding potential through molecular docking analysis. The comparative results showed that among all proteins, HEF is higher in prevalence throughout the analysis as a potential (human-derived) microRNAs target. The target-site conservation results showed that core nucleotide sequence in three different strains is responsible for potential miRNA binding to different viral strains. Further steps to use these microRNAs may lead to new therapeutic insights on fighting influenza virus infection.

## Introduction

Viral outbreaks are increasingly threatening public health (Asif et al., 2020). One of the most recurrent human affecting virsus is group of Influenza viruses. These viruses are the members of the orthomyxoviridae family which include A, B, C, and D viruses (Christensen et al., 2008). Influenza C virus often causes upper respiratory infections such as common cold, sinusitis, pharyngitis, epiglottitis and laryngotracheitis (Dasaraju and Liu 1996). Influenza C virus has an average lenght of >300 nm (filamentous rods) and is composed of seven segments of the genomic single-stranded RNAs (ssRNAs) (Pellett et al., 2014). The genomic segments range in length from ∼0.9 to ∼2.4 kb, with a genome bridged gene length of ∼14 kb. The viral particle is 80–120 nm in diameter so that small virions adopt an elliptical shape. Particle lengths vary widely and can exceed tens of micrometers; producing filamentous virions. The total genome size is 12,907 bp (Wagaman et al., 1989). Influenza C virion envelopes contain a single glycoprotein called hemagglutinin-esterase-fusion (HEF) protein. HEF is a typical 1 transmembrane protein with a short N-terminal, a smart signal peptide (14 amino acids), a long ectodomain (612 amino acids), a transmembrane region (26 amino acids) and a very short cytoplasmic tail (three amino acid long). The HEF present in the infectious membranes is made up of two subunits, the N-terminal containing 432 amino acids forming the HEF1 polypeptide, and a residual sequence including hydrophobic fusion peptide, a transmembrane domain (TMD) and a cytoplasmic tail called HEF2 (Wang and Veit 2016). It encapsulates RNA with a negative strand and protects it from nucleases. The synthetic genomic RNA is called ribonucleoprotein (RNP) and serves as a model for transcription and replication. Shortly after a virion infects a new cell, the matrix 1 (M1) protein breaks down the RNP during the acidification of the virion driven by the matrix 2 (M2) protein. The separation of M1 from the RNP emits nuclear localization signals, directing the RNP to the nucleus (Muraki and Hongo 2010).

Most importantly, viruses can stimulate their life cycle by altering the cellular environment by actively controlling the expression of many microRNA (miRNA) cells (Perez et al., 2009). miRNAs are small endogenous ssRNA molecules, 21–23 nucleotides in length, which are formed after processing of hairpin loop-like miRNA precursors (pre-miRNA) by an RNase-III-like enzyme (Dicer) and can regulate the gene expression through working in cohort with mRNA and related enzyme structures (Brodersen and Voinnet 2006; Ali et al., 2020). RNA silencing, through miRNAs present in humans, thus impairs natural immunity and resistance to the host against foreign genetic elements including influenza C virus (Li et al., 2004). In connection with the influenza C virus genome, 264 mature human miRNAs have been found (MacFarlane and R Murphy 2010); a subset of these mature miRNAs should have targets in the influenza C virus genome and these miRNAs, once identified, can be expressed through cloning to enhance the resistance against infection from viruses (Peng et al., 2018).

The goal of this research study was to implement the computational approaches for the identification of targets for human miRNAs in the influenza C virus genome, as a precedent for enhancing the resistance to influenza through RNA interference (RNAi). For this objective, a set of miRNAs was retrieved from miRNA databases and was tested for binding to the influenza C virus genome through in-silico experiments involving three different miRNA target sites prediction algorithms.

## Computational Methodology

### Retrieval of Genome Sequence and Annotations

The data was retrieved from the National Center for Biological Information (https://www.ncbi.nlm.nih.gov/) and the CLC Genome Workbench (v 9.5.2) was used to download the complete annotated genome sequence of influenza C virus with the accession numbers ranging from NC_006306.2 to NC_006312.2. The CLC sequence viewer was used to create alignment between different strains of influenza C virus proteins.

### Retrieval of Mature miRNA From miRBase

The miRBase (http://www.mirbase.org/) is a database that collects information on miRNAs sequences and their annotations (Saini et al., 2008; Kozomara and Griffiths-Jones 2014) and 264 human miRNAs were retrieved from miRBase.

### miRNA Target Sites Predictions

Three tools, RNA22 (Rigoutsos et al., 2007), miRanda (Enright et al., 2003) and RNAhybrid (Krüger and Rehmsmeier 2006) were employed for prediction of possible targets for human derived miRNAs in influenza C virus genome to locate miRNA targeting regions.

### RNA22

RNA22 (Miranda et al., 2006; Loher and Rigoutsos 2012) uses an approach different than the other miRNA prediction tools; it implements a pattern-based approach combined with estimation of the folding energy to locate the possible miRNA target sites without a cross-species conservation filter. Identification of putative miRNA target sites is also possible even without knowing the identity of the targeting miRNA. The algorithm first analyzes the sequences of known mature miRNAs and then based on pattern information from the miRNAs, predicts the putative target sites, with many aligned patterns and then identifies the miRNAs which are likely to bind to the predicted target sites. RNA22 was accessed from the web (cm.jefferson.edu/rna22/Interactive/) and the miRNAs and the target genome were input to the algorithm. Sensitivity and specificity values were kept at 63 and 61%, respectively and seed size of 7 was selected with one unpaired base allowed in the seed region without limitining the maximum number of G:U wobbles in the seed region. Minimum number of paired-up bases was kept at 12 while the maximum folding energy was kept at −14 kcal/mol.

### miRanda

The miRanda algorithm (John et al., 2004) has been used for miRNA target prediction (Zhao and Xue 2019). miRanda generally focuses on basic parameters such as sequence complementarity, free energy of RNA-RNA duplex and cross-species conservation of the target site to produce the output which is a weighted sum of match and mismatch scores for base pairs and gap penalties. Furthermore, it also promotes the prediction of multiple miRNA target sites including the ones with imperfect binding in the seed region within the 3′UTR of the target site thereby enhancing the specificity (Betel et al., 2008; M Witkos et al., 2011). The algorithm was run after defining the settings (gap open penalty = −9.0, gap extend penalty = −4.0, score threshold = 140, energy threshold = −20 kcal/mol and scaling parameter = 4.0).

### RNAhybrid

RNAhybrid is a tool for finding the minimum free energy for long and short miRNA. Hybridization is done in the form of domain mode, i.e. the short sequence is mixed within the most appropriate region of long sequences. miRNAs are short-acting RNAs that regulate gene targeting by binding to targeted mRNAs (Maziere and Enright 2007). Typically, RNAhybrid detects excellent hybridization sites of small RNA to large RNA. In RNAhybrid default parameters (minimum free energy value −30.0) were adjusted to predicts the miRNA targeting sites in influenza C virus. Morevoer, GUULGe tool was also employed to predict the miRNAs target sites (Gerlach et al., 2006).

### RStudio and Ggplot2

RStudio (https://rstudio.com/) is an integrated development environment (IDE) for R language usually employed for mathematical calculations and graphics. The ggplot2 package (https://cran.r-project.org/web/packages/ggplot2/index.html) was used to depict the graphical representations of miRNA predictions.

### miRNA and mRNA Model Predictions

MC-Fold (https://major.iric.ca/MC-Fold/) and MC-Sym (https://major.iric.ca/MC-Sym/) is an online pipeline to produce 3D models of miRNAs. The designed miRNA models were downloaded in PDB format and used for further structural analysis. For mRNA model prediction, RNAComposer, an online platform was employed to predict 3D structures of mRNA molecules (Biesiada et al., 2016). The mRNA sequence retrieved from NCBI database, was entered into RNAComposer to predict 3D structure of mRNA of HEF using default parameters. Moreover, UCSF Chimera, tool was used to visualize the miRNA and mRNA models and to depict their graphical images (Meng et al., 2006).

### Molecular Docking of Mature miRNA and mRNA

**FIGURE 1:**
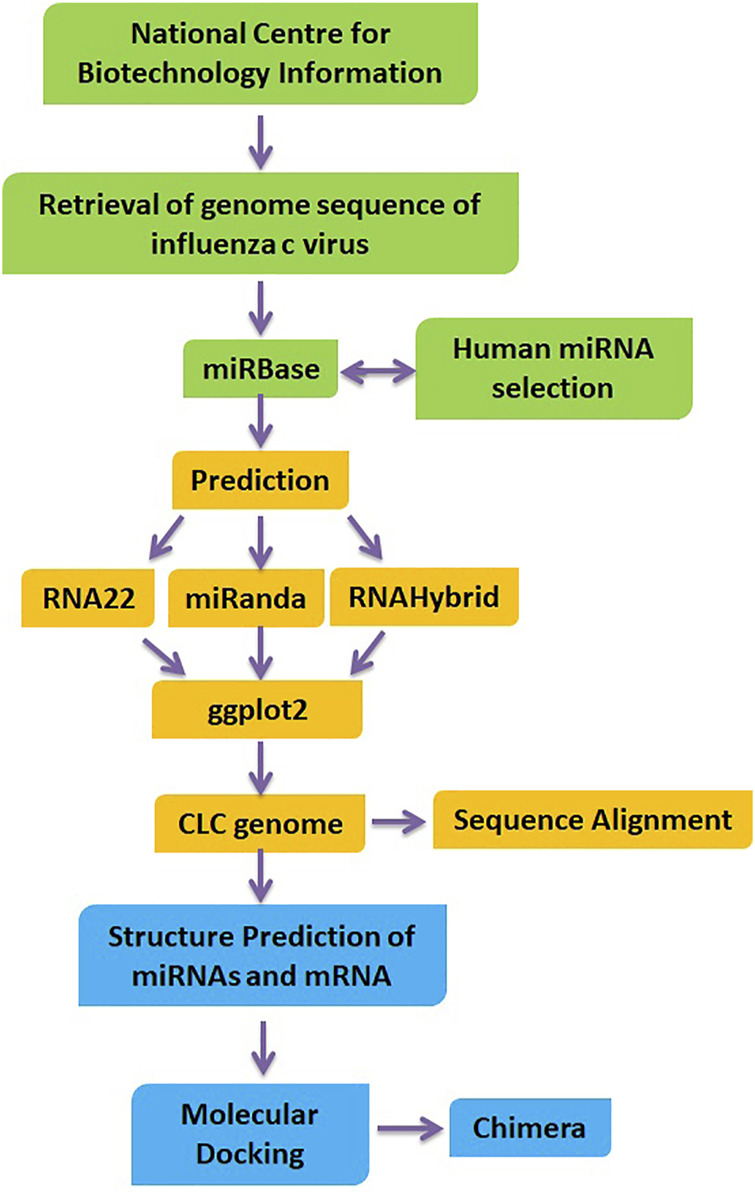
Flow chart of designed research work.

The PatchDock algorithm is a molecular docking algorithm based on shape complementarity principles (https://bioinfo3d.cs.tau.ac.il/PatchDock/). This method simultaneously addresses the problem of flexibility and scoring of models produced by fast rigid-body docking algorithms. We obtained 100 potential docking candidates; PatchDock refines and scores them according to an energy function, spending about 3.5 s per candidate pose. The bonding interaction patterns between miRNA and mRNA for predictions from all target prediction servers were analyzed by using Discovery Studio (4.1) and UCSF Chimera 1.10.1 (Meng et al., 2006), respectively. The overview of our proposed methodology has been illustrated in Figure 1.

## Results

**FIGURE 2:**
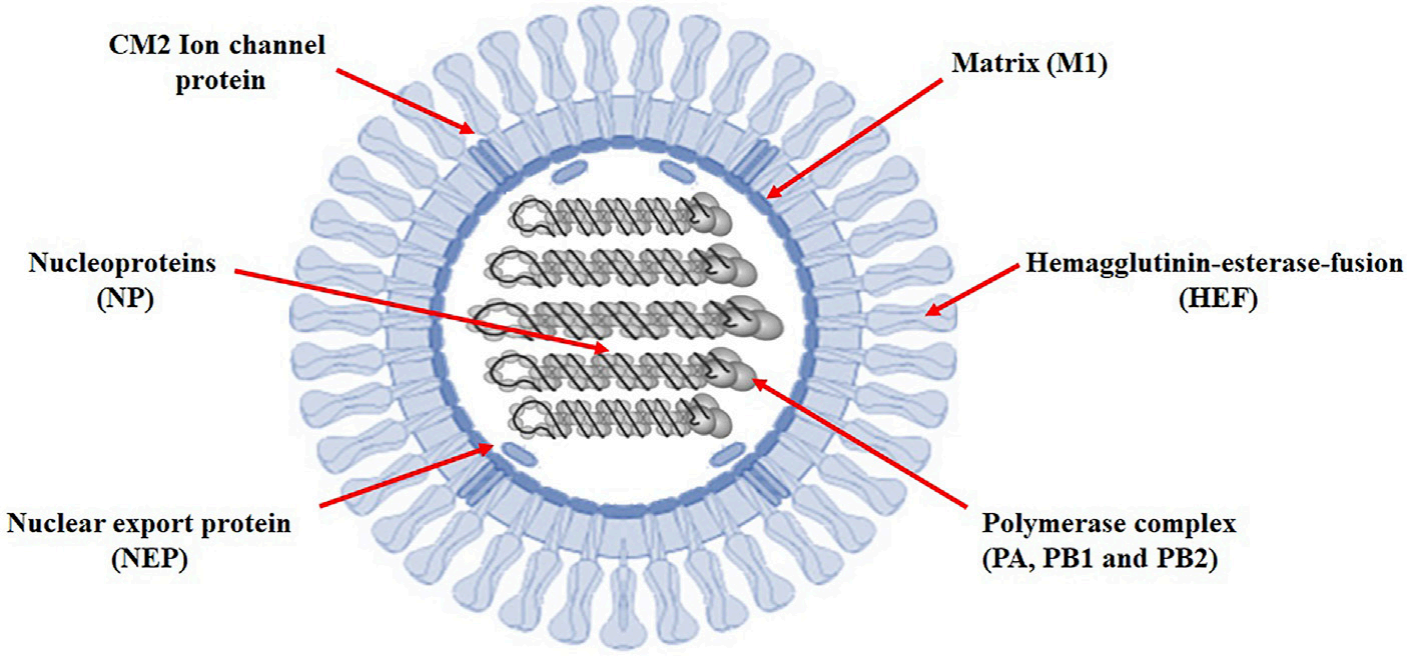
Influenza C virus genome. Prediction of new target-sites for miRNAs in influenza C virus.

Identifying the sequence of miRNAs is the first step in understanding the biology of miRNAs (Saif et al., 2019). Research and testing methods revealed many aspects of miRNA-target interactions and led to effective predictions of targeted mRNAs. Information collected on the recognition of the miRNA targets revealed the principles of miRNA-mRNA duplex formation, such as the binding of hsa-mir-3155a, hsa-mir-6796-5p, hsa-mir-3194-3p and hsa-mir-4673 to the influenza virus genome. Target selection of proteins based on predicted miRNAs would be important evidence of better understanding of the functionality of miRNAs and the prevalence of influenza C virus. All the miRNA-targets in influenze virus c genome are listed in supplementary data (*see* meta data). The genomic structure of influence c virus genome has been represented in Figure 2.

miRNAs are small double-stranded RNAs that exert a fine-tuning sequence-specific regulation in the cellular transcriptome (Zeng et al., 2003). miRNA regulates multiple mRNAs through binding with complementary sequences at 3′-untranslated regions for triggering the mechanism of RNA interference (Titze-de-Almeida et al., 2020). In our computational studies, target sites for 264 human-derived miRNAs from miRBase have been predicted and analyzed (hairpin and genomic location), based on microRNA-mRNA complementarity. The genomic position of four predicted miRNAs is hsa-mir-3155a (Chr10: 6152196-6152277); hsa-mir-6796-5p (Chr19: 40369846-40369907) hsa-mir-3194-3p (Chr20: 51452905-51452977) and hsa-mir-4673 (Chr9: 136519568-136519626), respectively.

### RNA22 Analysis

**FIGURE 3:**
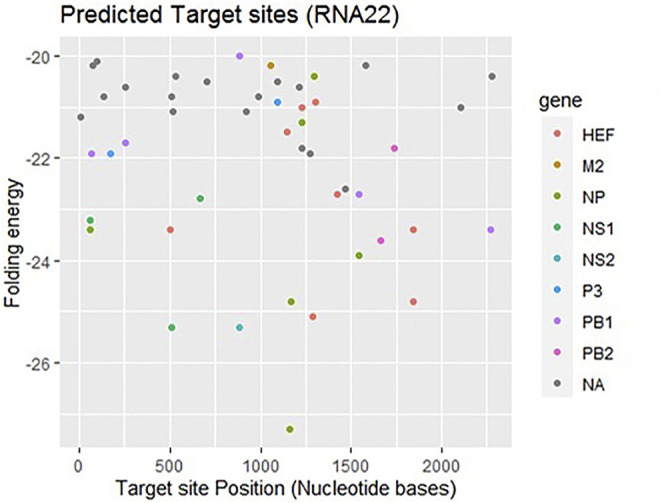
Target sites for miRNAs binding to influenza C virus genome predicted by RNA22 algorithm. The color dots represent binding to genes coding the relevant proteins. The gray dots labeled as NA which indicate non-applicable.

RNA22, a gene predictor uses a pattern-based approach and folding energy to find locations that can be targeted by miRNA without applying various types of conservation filters (Mazière and Enright 2007). Figure 3 shows that most frequently targeted sites where those with the folding energy value ranging from −20 to −22 kcal/mol and nucleotide position ranges from 0 to 2000 bp. Moreover, best predicted target sites belong to genes encoding proteins: HEF, M2, NP, NS1- NS2, P3, PB1, and PB2, respectively. The corresponding alignments have been shown in supplementary data (Supplementary Figure S1).

### miRanda

**FIGURE 4:**
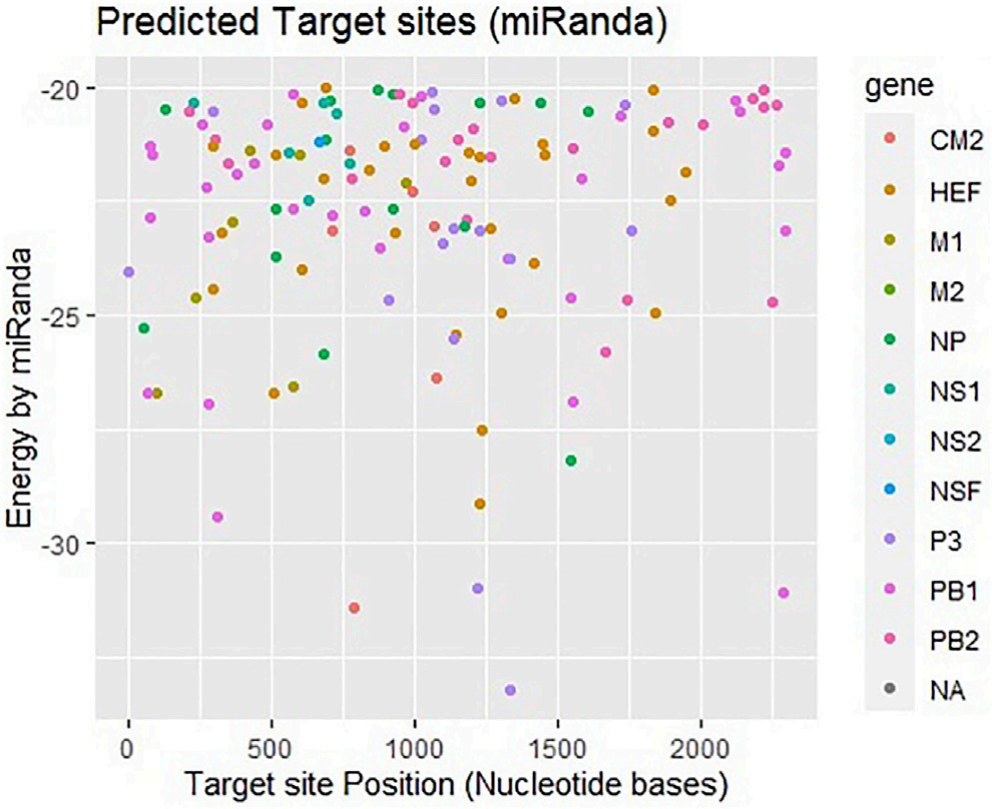
Target sites of miRNAs in genome of influenza C virus predicted by miRanda algorithm. The color key represents the respective genes/proteins in the graph. The gray dots labeled as NA which indicate non-applicable.

Another tool miRanda was employed and analyzed results based on miRNA-mRNA sequence similarity, free energy (ranging from −18 to −33 kcal/mol), and scoring values (from 140 to 185). Figure 4 shows that most frequently targeted sites are those where the free energy value ranges from −20 to −30 kcal/mol and nucleotide position range is 0–2000 bp. Moreover, significantly predicted target sites belong to genes encoding proteins CM2, HEF, M1-M2, NP, NS1- NS2, NSF, P3, PB1- PB2 and NA, respectively. The alignment figure has been reported in supplementary data (Supplementary Figure S1).

### RNA Hybrid

**FIGURE 5:**
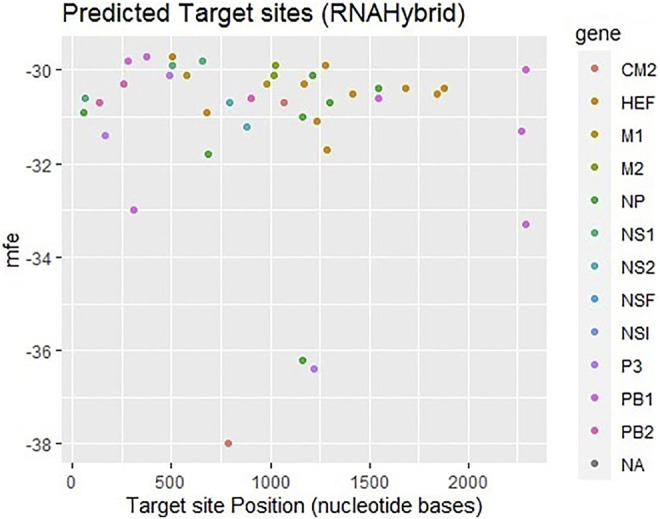
Target sites of human miRNAs in genome of influenza C virus predicted by RNAhybrid server. The color key represents the respective genes in the graph. The gray dots labeled as NA which indicate non-applicable.

The third approach which has been employed is RNAhybrid which identifies the regions in 30 UTRs which have the potential to form a thermodynamically favorable duplex with a specific miRNA. RNAhybrid uses minimum free energy (MFE) to evaluate the predicted binding sites for miRNAs. Figure 5 shows that most frequently targeted sites are those where the MFE ranges from −30 to −38 kcal/mol and nucleotide position range is 0–2000 bp. Moreover, best predicted target sites belong to genes encoding proteins CM2, HEF, M1-M2, NP, NS1- NS2, NSF, P3, PB1- PB2 and NA, respectively. The corresponding alignment (Supplementary Figure S1) has been reported in supplementary data.

**FIGURE 6:**
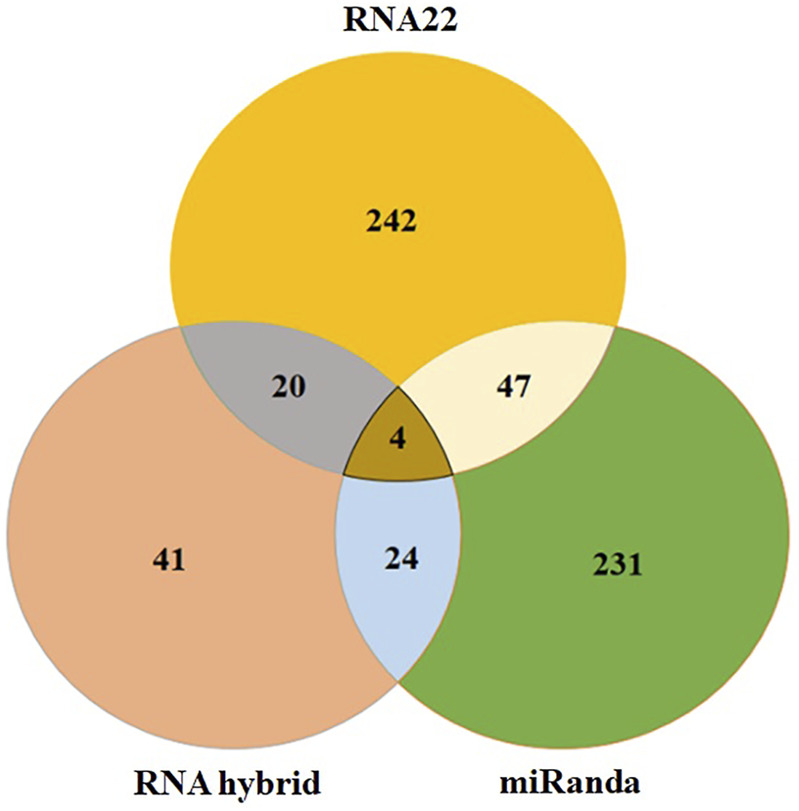
The Venn diagram of miRNAs predicted to bind to influenza C virus genomein by all three different tools: RNAhybrid, RNA22, and miRanda. The intersection graph showed four common miRNAs: hsa-mir-3155a, hsa-mir-6796-5p, hsa-mir-3194-3p and hsa-mir-4673, respectively.

RNA22, miRanda and RNAhybrid predicted 242, 231 and 41 miRNAs which may have possible binding sites against the influenza C virus genome and four most common hsa-mir-3155a, has-mir-6796-5p, has-mi3194-3p and has-mir-4673, respectively (Figure 6). Moreover, in union graph analysis 47 miRNAs were common in RNA22-miRanda predictions, 20 and 24 miRNAs were common in RNA22-RNAhybrid and RNAhybrid-miRanda predictions, respectively (Figure 6). The common features of all three algorithms have been reported in Table 1 (Peterson et al., 2014). Furthermore, GUUGLe predicted results showed less consensus to pur predicted results and therefore, generated result has been tabulated in supplementary data (Meta Data, sheet 3).

**TABLE 1:** Common features of miRanda, RNA22 and RNAhybrid algorithms.

Algorithms	Seed match	Conservation	Free energy	Target-site abundance
miRanda	Yes	Yes	Yes	—
RNA22	Yes	—	Yes	—
RNAhybrid	Yes	—	Yes	Yes

### Prediction of Structures of hsa-mir-4673 and hsa-mir-3194-3p and Its Cellular Functionality

**FIGURE 7:**
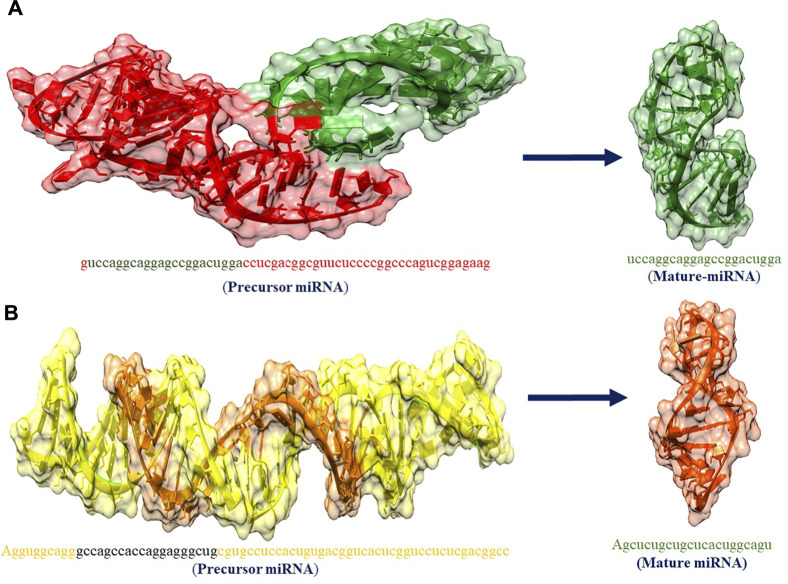
**(A**,**B)** The predicted 3D structures of premature and mature has_mir_4673 and has_mir_3194_3p miRNAs, respectively.

The hsa-mir-4673 and hsa-mir-3194-3p play an important role in our studies. The miRNA acts as a guide for basic pairing with the target mRNA to negatively control its expression. The paired miRNAs are aimed at mRNA targeting gene mutation by mRNA cleavage or translation compression based on the degree of compatibility between miRNA and mRNA target. Figure 7 shows the predicted 3D structures of premature and mature hsa-mir-4673 and hsa-mir-3194-3p.

### Prediction of Structures of hsa_mir_6796-5p and hsa-mir-3155a and Their Cellular Functionality

**FIGURE 8:**
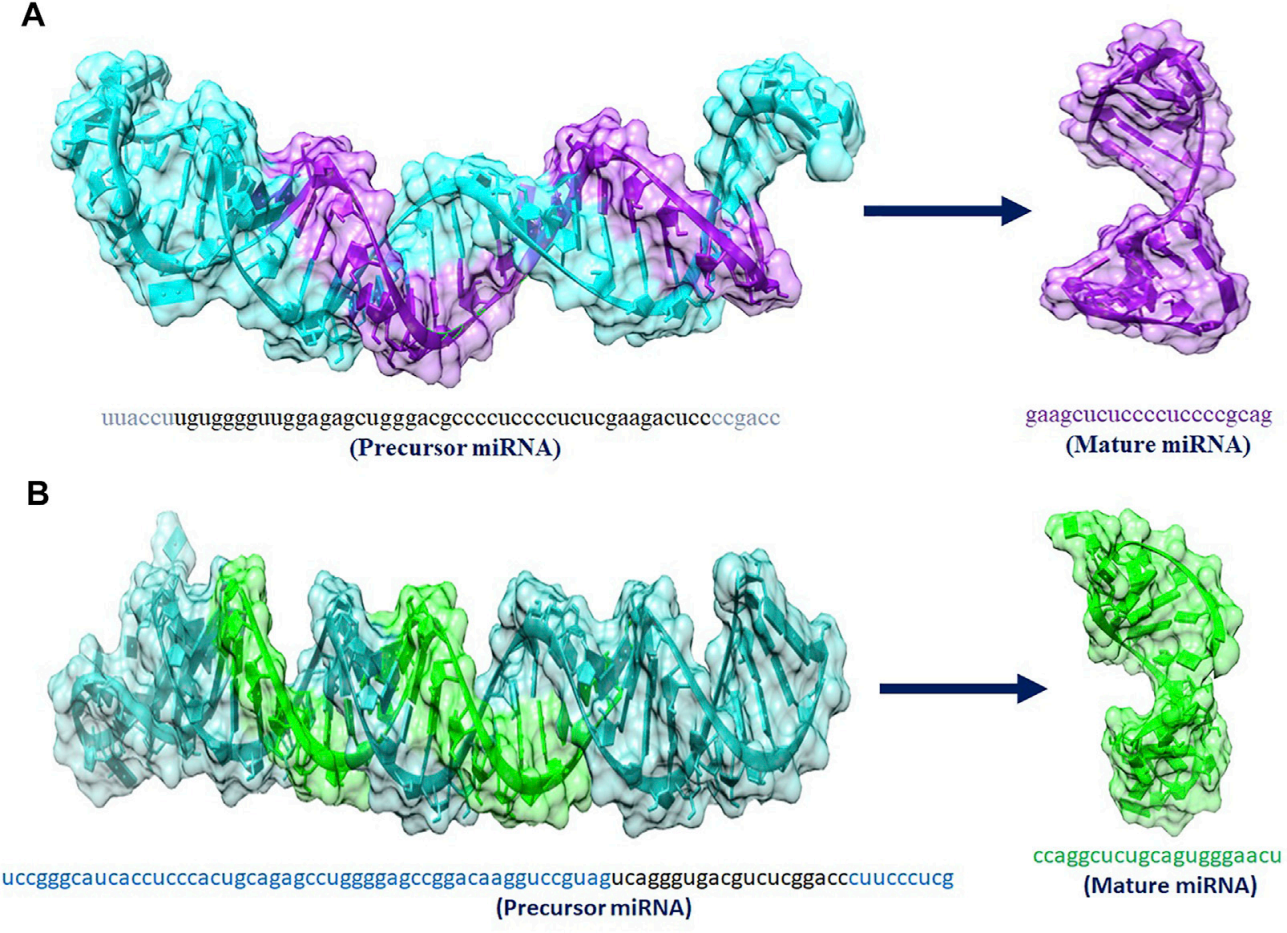
**(A**,**B)** 3D structures of premature and mature miRNAs hsa_mir_6796-5p and has_mir_3155a, miRNAs respectively.

The hsa_mir_6796_5p and hsa_mir_3155a are playing an important role in our studies. miRNAs are short non-coding RNAs that regulate gene expression after transcription. They usually bind 3′-UTR (uninterrupted region) of their targeted mRNAs and suppress protein production by damaging mRNA and silencing translation. Figure 8 shows the predicted 3D structures of premature and mature hsa-mir-6796-5p and hsa-mir-3155a.

### Target-Site Conservation Analysis

**FIGURE 9:**
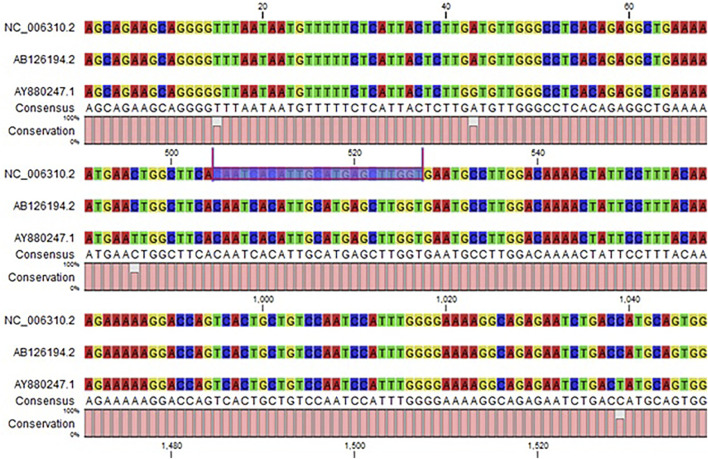
Target-site conservation pattern in different influenza strains. The target sites are highlighted in blue, and bars show the level of conservation at specific sites. Involvement of predicted proteins in influenza c virus.

Conserved miRNAs target sites tend to be enriched at the 5′ end of protein-coding gene 3′ UTRs and provide evidence that miRNA targets sites are enriched in genes involved in protein synthesis pathways (Xiong et al., 2019). Our results showed the conserved nucleotides in different influenza virus strains. This conservation pattern enhanced the accuracy and reliability of predicted miRNAs across the other strains (*see* Figure 9 and Supplementary Figures S3, S4).

### Hemagglutinin-Esterase-Fusion

HEF is composed of 655 amino acids (https://www.uniprot.org/uniprot/P07975). The HEF is divided into couple of subunits HEF1 and HEF2 through proteolytic cleavage (Wang and Veit 2016). HEF is a glycoprotein that is involved in receptor binding, receptor destroying and membrane fusion activities. HEF, recognizes and attaches to a receptor through determinant N-acetyl-9-O-acetylneuraminic acid on the cell surface and initiates virus entry (Rogers et al., 1986). Moreover, HEF also catalyzes the fusion of the viral envelope with endocytic vesicles. Finally, HEF is the receptor-destroying enzyme, which is the function of neuraminidase (NA) in the influenza A and B viruses. HEF does not cleave the terminal sialic acid residue from carbohydrates but has an esterase activity that removes the acetyl group from position C-9 of N-acetyl-9-Oacetylneuraminic acid (Herrler et al., 1985). This function is probably required to release freshly budded virus particles from infected cells, which would otherwise be trapped at their plasma membrane if the receptor would still be present (Wang and Veit 2016).

### Nucleoprotein (NP)

The NP containing 498 amino acids is a highly conserved multi-functional viral protein, best known as a hot antiviral drug target. It has been observed that upon attachment to the host cell through the interaction between HA from the virus and the sialic acid receptor on the host cell surface, the influenza virus enters host cells by endocytosis (Hu et al., 2017). The X-ray crystal structures of the influenza nucleoprotein are known, the pockets in which these mutants (Y289H, N309K/N309T, and Y52H) reside might be the corresponding drug binding sites of nucleozin (Kao et al., 2010).

### Solute Carrier Family 10 Member 3

Solute Carrier Family 10 Member 3, also known as P3 protein contains 716 amino acids. It has been observed that active P3 (coded by RNA 1) and P1 protein (coded by RNA 2) are required for complementary RNA synthesis of influenza virus (Yamashita et al., 1989). Therefore, this protein could be used as a novel target to design chemical inhibitors to stop the virulence of influenza C virus.

### Polymerase Basic Protein 1 and 2

**FIGURE 10:**
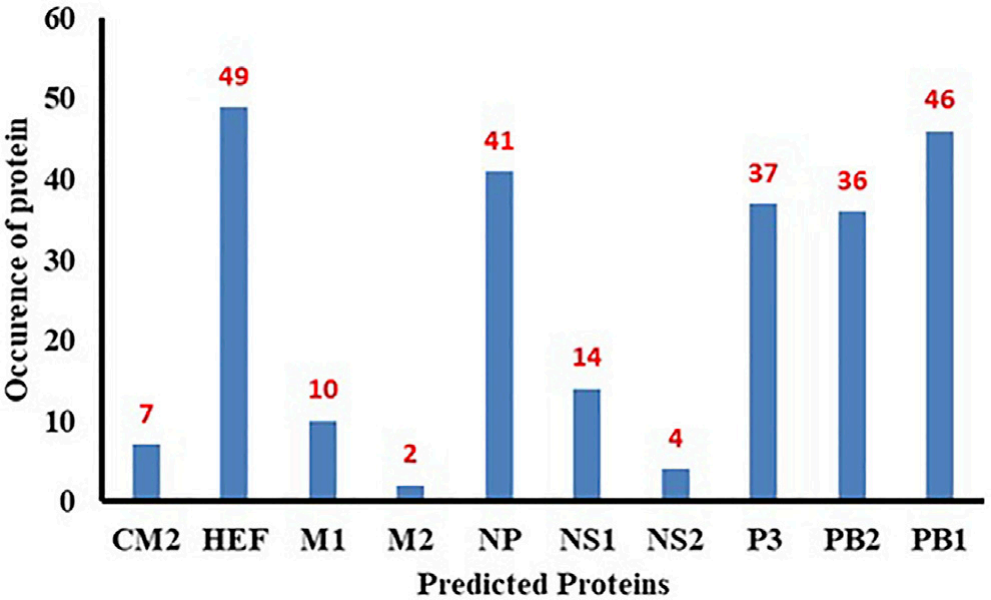
Bar chart of predicted proteins.

The PB1 protein consists of 757 amino acids, occurs in influenza viruses A, B and C and is involved in the RNA elongation and endonuclease activity (Bouvier and Palese 2008). The PB2 genecontains 2341 nucleotides and codes 759 amino acids-long PB2 protein that is responsible for mRNA cap recognition (Bouvier and Palese 2008). All influenza viruses encode the polymerase subunit PB1 on segment 2; in some strains of influenza A virus, this segment also codes the accessory protein PB1-F2, a small, 87-amino acid protein with pro-apoptotic activity, in a +1 alternate reading frame (Chen et al., 2001). The predicted frequency of occurrence of all proteins has been shown in Figure 10.

### Molecular Docking Analysis

**FIGURE 11:**
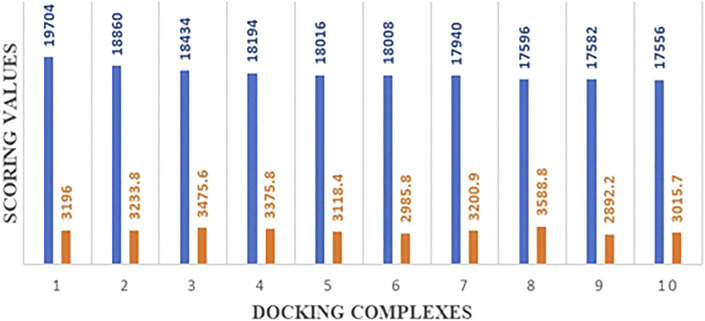
Docking energy values of top 10 best complexes.

The predicted 3D structures of hsa-miRNA-3155a and mRNA of HEF were docked to analyze the binding potential and nucleotide region involved in the binding conformation. The docking results showed that a good scoring value has been observed with better interactive behavior. The best docking score was 19,704, which demonstrates very good geometric shape complementarity. Moreover, the approximate interface area and atomic contact energy of docked complex were 3196 and −2078.92, respectively (Figure 11). The detailed docking scoring values have been tabulated in supplementary data (Supplementary Table S1).

**FIGURE 12:**
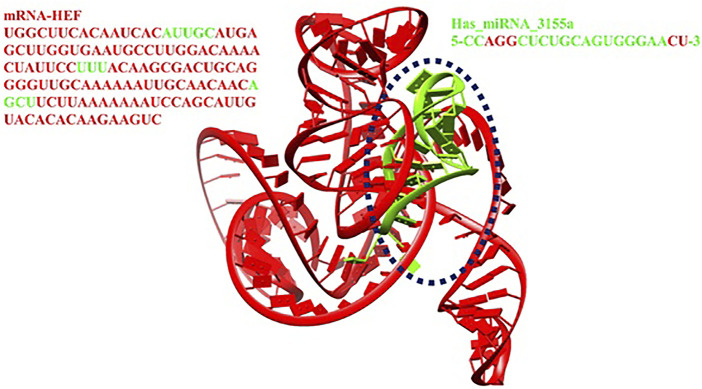
Docking complex of hsa-miRNA-3155a.

The interactive behavior of hsa-miRNA-3155a and mRNA of HEF was clearly observed and generated results show that multiple nucleotides of miRNA-3155a form close contacts with mRNA of HEF. The nucleotide adenine, guanine and guanine (AGG) at positions 3, 4 and 5 in miRNA-3155a formed close interaction with mRNA of HEF. Similarly, cytosine and uracil (CU) at positions 20 and 21 also showed close interactions with mRNA of HEF. Similarly, different regions in mRNA-HEF such as AUUGC, UUU and AGCU have been predicted as targets for potential binding with hsa-miRNA-3155a (Figure 12). The docking results showed that binding of hsa-miRNA-3155a with mRNA-HEF may stop the translation process and can protect from influenza C virus infection.

### Mechanistic Pathways of miRNAs

**FIGURE 13:**
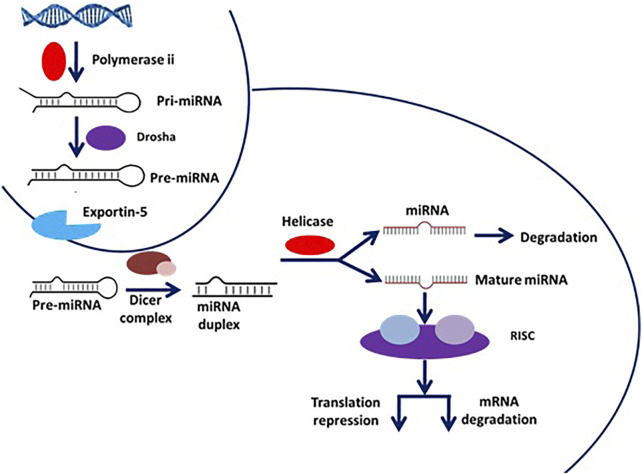
Mechanistic pathway of miRNA formation from premature miRNA.

miRNAs are small, highly conserved non-coding RNA molecules (containing about 22 nucleotides) involved in the regulation of differential gene expression. miRNAs are transcribed by RNA polymerases II and III, generating precursors that undergo a series of cleavage events to form mature miRNA (MacFarlane and R Murphy 2010). The pri-miRNA (primary transcript) is converted into pre-miRNA (precursor-miRNA, a stem-loop structure of about 70 base pairs long) using DROSHA, a nuclear RNase III enzyme that executes the initiation step of miRNA processing in the nucleus (Lee et al., 2003) (Nguyen et al., 2018). The overall mechanistic pathway of mature miRNA formation has been depicted in Figure 13.

### miRNAs and Immune Response

**FIGURE 14:**
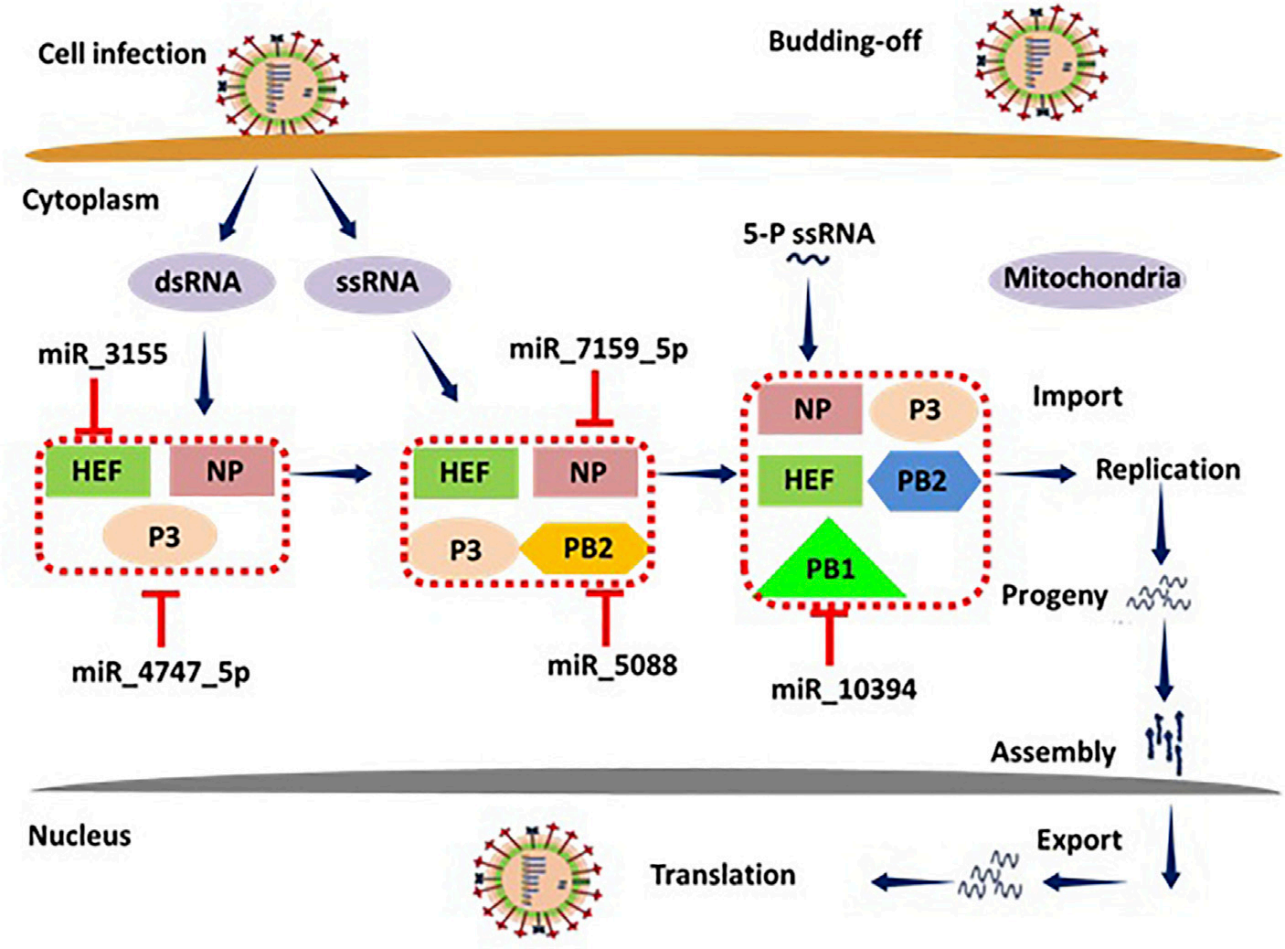
Mechanistic pathway of miRNAs involvement in the immune response.

miRNAs regulate the immune responses (both acquired and innate immunity) against flu infection by activating the different protein signaling cascades in humans (Zhang and Li 2013). During infection, a variety of intracellular signaling pathways are being activated at different receptor sites such as TNF receptor-related substances (TRAFs), and interferon-regulatory features (IRFs) through different proteins HEF, P3, NP, PB1 and 2, CM1 and 2, NS1 and 2, respectively. The HEF is involved in the influenza (Wang and Veit 2016), therefore miRNA-3155 binding to HEF may inhibit the downstream signaling pathway (*see* Figure 14). Moreover, PB1 and PB2 are also key players in the activated signaling pathways after the incorporation of pathogen within the host cells (Ludwig 2011). Our computational results show that miRNA-5088 has the potential to bind to complementary sites of mRNA of PB2 and stop the further transcriptional process. As a result, miRNA-5088 may play a supportive role in the prevention of influenza. Other miRNAs such as miRNA-7159, miRNA-4747 and miRNA-10394 bind to NP, P3 and PB1, respectively and may stop their signaling cascades and therefore could be used as good protective agents against influenza infection (Gaur et al., 2011). The overall mechanistic pathway of miRNA involvement in the immune response is shown in Figure 14.

## Discussion

In the current study, we have designed an effective computational approach for the validation of miRNA target prediction results by utilizing RNA22, miRanda and RNAhybrid different algorithms with default parameters. These algorithms proposed an effective method to recognize the possible host-derived miRNA targets in virus genomes (Ashraf et al., 2020; Ashraf et al., 2022). The RNA22 is an algorithm for exploring new miRNA-mRNA interactions because of its unique capabilities-although it has a high likelihood of generating false-positive results (Riffo-Campos Á.L et al., 2016). miRanda is mostly extensively used algorithm that includes the main aspects of miRNA-target prediction, such as the conservation level and miRNA 3′UTR site (Betel D et al., 2010). RNAhybrid is also algorithm to identify the minimum free energy for long and short miRNAs (Maziere and Enright 2007).

In our computational results, target sites for 264 human-derived miRNAs from miRbase have been predicted and analyzed (hairpin and genomic location), based on miRNA-mRNA complementarity. The comparative results showed that a total of 242, 231 and 41 miRNAs have been predicted to bind with influenza C virus genome by RNA22, miRanda and RNAhybrid, respectively. In union graph analysis among all three utilized algorithms, we have observed 47 common miRNAs inbetween RNA22-miRanda predictions, whereas 20 and 24 miRNAs common in RNA22-RNAhybrid and RNAhybrid-miRanda predictions, respectively. In intersection graph analysis, we have found that four miRNAs: hsa-mir-3155a, hsamir-6796-5p, hsami3194-3p and hsamir-4673 which were common in three algothims methods which suggest their good binding potential with targeted sites in influenza C virus genome (Figure 6; Table 2).

**TABLE 2:** Premature and mature miRNAs.

miRNAs	Precursor miRNAs	Mature miRNAs
hsa-mir-3155a	ucc​ggg​cau​cac​cuc​cca​cug​cag​agc​cug​ggg​agc​cgg​aca​agg​ucc​gua​guc​agg​gug​acg​ucu​cgg​acc​cuu​ccc​ucg	Cca​ggc​ucu​gca​gug​gga​acu
hsa-mir-6796-5p	uua​ccu​ugu​ggg​guu​gga​gag​cug​gga​cgc​ccc​ucc​ccu​cuc​gaa​gac​ucc​ccg​acc	Uug​ugg​uug​gag​agc​ugg​c
hsa-mir-3194-3p	Agg​ugg​cag​ggc​cag​cca​cca​gga​ggg​cug​cgu​gcc​ucc​acu​gug​acg​guc​acu​cgg​ucc​ucu​cga​cgg​cc	Agc​ucu​gcu​gcu​cac​ugg​ca
hsa-mir-4673	guc​cag​gca​gga​gcc​gga​cug​gac​cuc​gac​ggc​guu​cuc​ccc​ggc​cca​guc​gga​gaa​g	Ucc​agg​cag​gag​ccg​gac​ugg​a

The minimum free energy (MEF) structure of a sequence is the secondary structure that is calculated to have the lowest value of free energy. Free energy calculation is a dynamic feature of miRNA and target binding. Prior research data showed a significant correlation of free energy between the translational repression and the hybridization binding of the seed region (Doench J.G. and Sharp PA, 2004). The thermodynamic stability of the miRNA-mRNA duplex was estimated by the assessment of free energy to monitor site accessibility for the determination of the secondary structure duplex (Peterson et al., 2014). From RNA22 results, the predicted results were analyzed based on the sensitivity (25%) and specificity (90%) and folding energy pattern. It has been observed that highest negative value of MEF is showed the most structured and stable miRNA-mRNA complexes. The most frequently targeted sites where those with the folding energy value ranging from −20 to −22 Kcal/mol and nucleotide position ranges from 0 to 2000 bp. Moreover, from miRanda results, it has also been observed a good binding site in with good sequence similarity, free energy, and scoring values (from 140 to 185), respectively. The best targeted sites have been observed where the free energy value ranges from −20 to −30 Kcal/mol with nucleotides range from 0–2000 bp. RNAhybrid identifies the regions in 30 UTRs which have the potential to form a thermodynamically favorable duplex with a specific miRNA. From RNAhybrid result, it has been observed that MFE ranges from −30 to −38 kcal/mol and nucleotide position range is 0–2000 bp. Moreover, in protein comparison results, we have identified multiple proteins: CM2, HEF, MI, M2, NP, NS1, NS2, P3, PB1 and PB2 that may have a significant role in cell signaling and functionality of genomic virus (Furukawa et al., 2011). It has been observed that CM2 is directly involved in the influenza C virus replication cycle (Furukawa et al., 2011). HEF showed the highest value (<50) as compared to rest of proteins whereas, M2 depicted the lowest value (<10). Moreover, NP, P3, PB2 and PB1 showed 41, 38, 46 and 37, respectively (Figures 7, 8). The genomic region has been mentioned in Supplementary Figure S2. The conserved nucleotides of showed the common predicting binding site for miRNAs among different strains of influenza virus (*see* Figure 9 and Supplementary Figure S3).

Molecular docking is a computational approach usually employed to check the binding potential of biomolecules (Hassan et al., 2022, 2021). The modelled 3D structures of hsa-miRNA-3155a and mRNA were docked to analyze the binding potential and nucleotide region involved in the binding conformations. The comparison results from all docking scores, it has been seen that 19704 was best docking score, which demonstrates very good geometric shape complementarity of miRNA against mRNA (*see* Figure 11). Furthermore, a detail conformation analysis was done to explore the nucleotides of miRNA having good binding interactions with mRNA. Our predicted results showed that adenine, guanine, and guanine (AGG) at positions 3, 4 and 5 in miRNA-3155a formed good interaction with mRNA of HEF. Similarly, cytosine and uracil (CU) at positions 20 and 21 formed hydrogen bonds with mRNA of HEF (Figure 12). The docking results showed that binding of hsa-miRNA-3155a with mRNA-HEF may stop the translation process and can protect from influenza virus infection.

## Conclusion

This study presents a planned *in silico* approach for finding host-derived miRNAs aimed at silencing the genome of influenza virus. Predicted results showed that miRNAs: hsa-mir-3155a, hsa-mir-6796-5p, hsa-mir-3194-3p and hsa-mir-4673 demonstrated good binding complementarity to influenza genome. Based on interactive behavior multiple proteins were identified as are active targets for influenza mediating signaling cascade in humans. Among all, HEF was repeatedly predicted in our computational studies. The docking results showed the binding pattern of hsa-mir-3155a to mRNA of HEF protein which also depicts the interactive nucleotides which may involves in the transcriptional activity HEF gene. However, molecular biology techniques are required to further verify our computational results. The target-site conservation pattern in different strains ensures the significance of hsa-mir-3155a. Taken together, exploration of these small endogenous nucleotides (miRNAs) may lead to new therapeutics against influenza virus infection.

## Data Availability

The original contributions presented in the study are included in the article/Supplementary Material, further inquiries can be directed to the corresponding authors.
