# Supplementary material for: Prediction of Site Directed miRNAs as Key Players of Transcriptional Regulators Against Influenza C Virus Infection Through Computational Approaches
Source: Front Mol Biosci. 2022 Apr 8;9:866072. doi: 10.3389/fmolb.2022.866072 (PMC9023806; doi:10.3389/fmolb.2022.866072)
Supplement: Supplementary file 1 [file DataSheet2.pdf]

Supplementary Material

Prediction of site directed miRNAs as key players of transcriptional regulators against influenza C virus infection through computational approaches

Mubashir Hassan[1,2*] Muhammad Shahzad Iqbal[3], Sawaira Naqvi[1], Hany Alashwal[*4] Ahmed A. Moustafa [5,6] Andrzej Kloczkowski[2,7*]

[1]Institute of Molecular Biology and Biotechnology, The University of Lahore (Defense Road Campus), Lahore, Pakistan.

[2]The Steve and Cindy Rasmussen Institute for Genomic Medicine, Nationwide Children Hospital, Columbus, Ohio-43205, USA.

[3]Department of Biotechnology, Faculty of Life Sciences, University of Central Punjab, Lahore, Pakistan.

[4]College of Information Technology, United Arab Emirates University, Al-Ain, 15551, UAE.

[5]Department of Human Anatomy and Physiology, the Faculty of Health Sciences, University of Johannesburg, South Africa.

[6]School of Psychology, Faculty of Society and Design, Bond University, Gold Coast, Queensland, Australia.

[7]Department of Pediatrics, The Ohio State University, Columbus, Ohio-43205, USA

Correspondence

Dr. Mubashir Hassan

Email: mubashirhassan_gcul@yahoo.com

Dr. Hany Alashwal

College of Information Technology, United Arab Emirates University, Al-Ain, 15551, UAE.

Email: halashwal@uaeu.ac.ae

Prof. Andrzej Kloczkowski

Email: Andrzej.kloczkowski@nationwidechildrens.org

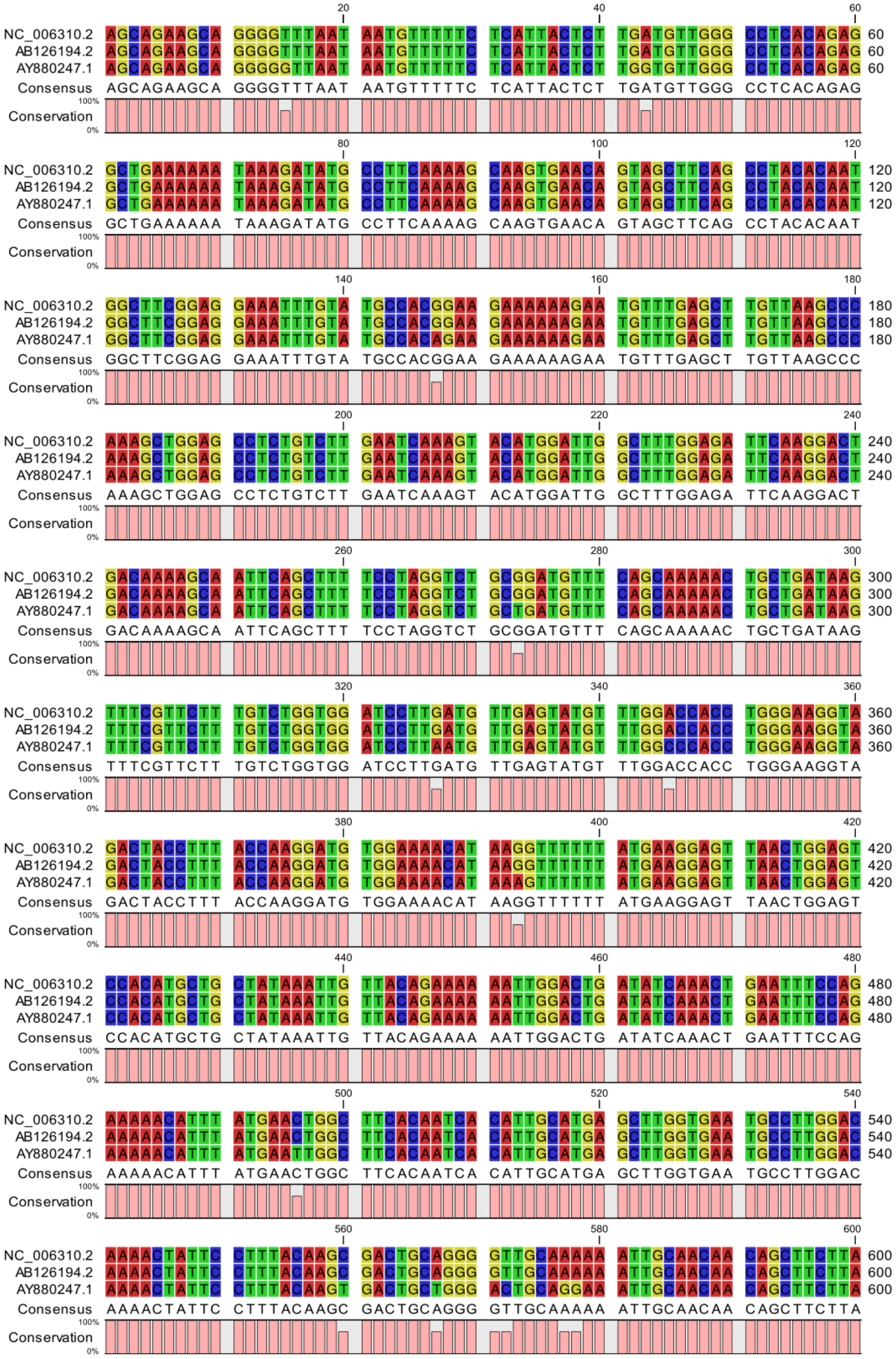
20 40 60
NC_006310.2 AGCAGAAGCA GGGGTTTAAT AATGTTTTTC TCATTACTCT TGATGTTGGG CCTCACAGAG 60
AB126194.2 AGCAGAAGCA GGGGTTTAAT AATGTTTTTC TCATTACTCT TGATGTTGGG CCTCACAGAG 60
AY880247.1 AGCAGAAGCA GGGGGTTAAT AATGTTTTTC TCATTACTCT TGGTGTTGGG CCTCACAGAG 60
Consensus AGCAGAAGCA GGGGTTTAAT AATGTTTTTC TCATTACTCT TGATGTTGGG CCTCACAGAG
Conservation 100% 0%
80 100 120
NC_006310.2 GCTGAAAAAA TAAAGATATG CCTTCAAAAG CAAGTGAACA GTAGCTTCAG CCTACACAAT 120
AB126194.2 GCTGAAAAAA TAAAGATATG CCTTCAAAAG CAAGTGAACA GTAGCTTCAG CCTACACAAT 120
AY880247.1 GCTGAAAAAA TAAAGATATG CCTTCAAAAG CAAGTGAACA GTAGCTTCAG CCTACACAAT 120
Consensus GCTGAAAAAA TAAAGATATG CCTTCAAAAG CAAGTGAACA GTAGCTTCAG CCTACACAAT
Conservation 100% 0%
140 160 180
NC_006310.2 GGCTTCGGAG GAAATTTGTA TGCCACGGAA GAAAAAAGAA TGTTTGAGCT TGTTAAGCCC 180
AB126194.2 GGCTTCGGAG GAAATTTGTA TGCCACGGAA GAAAAAAGAA TGTTTGAGCT TGTTAAGCCC 180
AY880247.1 GGCTTCGGAG GAAATTTGTA TGCCACAGAA GAAAAAAGAA TGTTTGAGCT TGTTAAGCCC 180
Consensus GGCTTCGGAG GAAATTTGTA TGCCACGGAA GAAAAAAGAA TGTTTGAGCT TGTTAAGCCC
Conservation 100% 0%
200 220 240
NC_006310.2 AAAGCTGGAG CCTCTGTCTT GAATCAAAGT ACATGGATTG GCTTTGGAGA TTCAAGGACT 240
AB126194.2 AAAGCTGGAG CCTCTGTCTT GAATCAAAGT ACATGGATTG GCTTTGGAGA TTCAAGGACT 240
AY880247.1 AAAGCTGGAG CCTCTGTCTT GAATCAAAGT ACATGGATTG GCTTTGGAGA TTCAAGGACT 240
Consensus AAAGCTGGAG CCTCTGTCTT GAATCAAAGT ACATGGATTG GCTTTGGAGA TTCAAGGACT
Conservation 100% 0%
260 280 300
NC_006310.2 GACAAAAGCA ATTCAGCTTT TCCTAGGTCT GCGGATGTTT CAGCAAAAAC TGCTGATAAG 300
AB126194.2 GACAAAAGCA ATTCAGCTTT TCCTAGGTCT GCGGATGTTT CAGCAAAAAC TGCTGATAAG 300
AY880247.1 GACAAAAGCA ATTCAGCTTT TCCTAGGTCT GCTGATGTTT CAGCAAAAAC TGCTGATAAG 300
Consensus GACAAAAGCA ATTCAGCTTT TCCTAGGTCT GCGGATGTTT CAGCAAAAAC TGCTGATAAG
Conservation 100% 0%
320 340 360
NC_006310.2 TTTCGTTCTT TGTCTGGTGG ATCCTTGATG TTGAGTATGT TTGGACCACC TGGGAAGGTA 360
AB126194.2 TTTCGTTCTT TGTCTGGTGG ATCCTTGATG TTGAGTATGT TTGGACCACC TGGGAAGGTA 360
AY880247.1 TTTCGTTCTT TGTCTGGTGG ATCCTTAATG TTGAGTATGT TTGGCCCACC TGGGAAGGTA 360
Consensus TTTCGTTCTT TGTCTGGTGG ATCCTTGATG TTGAGTATGT TTGGACCACC TGGGAAGGTA
Conservation 100% 0%
380 400 420
NC_006310.2 GACTACCTTT ACCAAGGATG TGGAAAACAT AAGGTTTTTT ATGAAGGAGT TAACTGGAGT 420
AB126194.2 GACTACCTTT ACCAAGGATG TGGAAAACAT AAGGTTTTTT ATGAAGGAGT TAACTGGAGT 420
AY880247.1 GACTACCTTT ACCAAGGATG TGGAAAACAT AAAGTTTTTT ATGAAGGAGT TAACTGGAGT 420
Consensus GACTACCTTT ACCAAGGATG TGGAAAACAT AAGGTTTTTT ATGAAGGAGT TAACTGGAGT
Conservation 100% 0%
440 460 480
NC_006310.2 CCACATGCTG CTATAAATTG TTACAGAAAA AATTGGACTG ATATCAAACT GAATTTCCAG 480
AB126194.2 CCACATGCTG CTATAAATTG TTACAGAAAA AATTGGACTG ATATCAAACT GAATTTCCAG 480
AY880247.1 CCACATGCTG CTATAAATTG TTACAGAAAA AATTGGACTG ATATCAAACT GAATTTCCAG 480
Consensus CCACATGCTG CTATAAATTG TTACAGAAAA AATTGGACTG ATATCAAACT GAATTTCCAG
Conservation 100% 0%
500 520 540
NC_006310.2 AAAAACATTT ATGAACTGGC TTCACAATCA CATTGCATGA GCTTGGTGAA TGCCTTGGAC 540
AB126194.2 AAAAACATTT ATGAACTGGC TTCACAATCA CATTGCATGA GCTTGGTGAA TGCCTTGGAC 540
AY880247.1 AAAAACATTT ATGAATTGGC TTCACAATCA CATTGCATGA GCTTGGTGAA TGCCTTGGAC 540
Consensus AAAAACATTT ATGAACTGGC TTCACAATCA CATTGCATGA GCTTGGTGAA TGCCTTGGAC
Conservation 100% 0%
560 580 600
NC_006310.2 AAAACTATTC CTTTACAAGC GACTGCAGGG GTTGCAAAAA ATTGCAACAA CAGCTTCTTA 600
AB126194.2 AAAACTATTC CTTTACAAGC GACTGCAGGG GTTGCAAAAA ATTGCAACAA CAGCTTCTTA 600
AY880247.1 AAAACTATTC CTTTACAAGT GACTGCTGGG ACTGCAGGAA ATTGCAACAA CAGCTTCTTA 600
Consensus AAAACTATTC CTTTACAAGC GACTGCAGGG GTTGCAAAAA ATTGCAACAA CAGCTTCTTA
Conservation 100% 0%

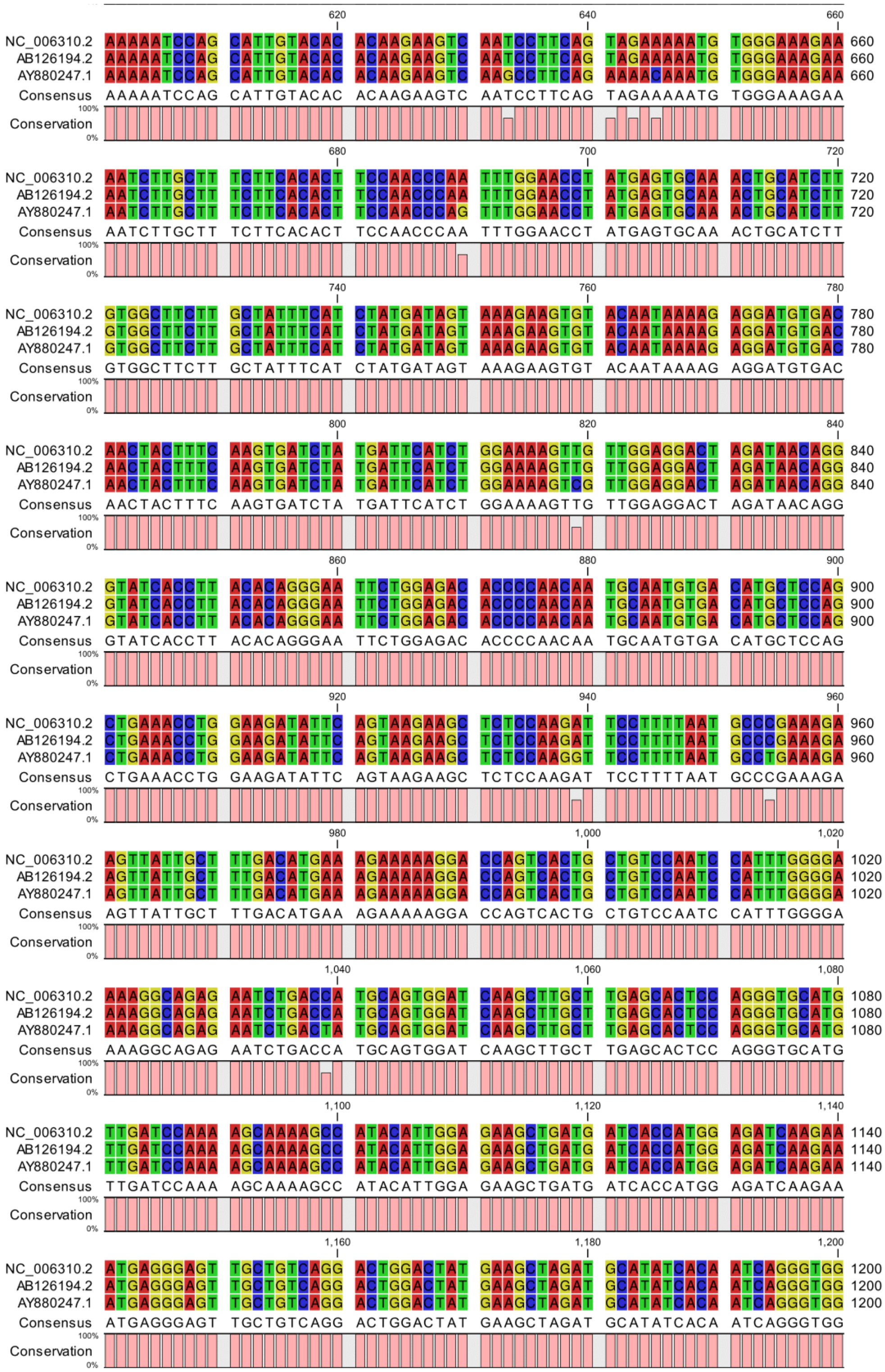

620 640 660
NC_006310.2 AAAAATCCAG CATTGTACAC ACAAGAAGTC AATCCTTCAG TAGAAAAATG TGGGAAAGAA 660
AB126194.2 AAAAATCCAG CATTGTACAC ACAAGAAGTC AATCCTTCAG TAGAAAAATG TGGGAAAGAA 660
AY880247.1 AAAAATCCAG CATTGTACAC ACAAGAAGTC AAGCCTTCAG AAAACAAATG TGGGAAAGAA 660
Consensus AAAAATCCAG CATTGTACAC ACAAGAAGTC AATCCTTCAG TAGAAAAATG TGGGAAAGAA
Conservation 100% 0%
680 700 720
NC_006310.2 AATCTTGCTT TCTTCACACT TCCAACCCAA TTTGGAACCT ATGAGTGCAA ACTGCATCTT 720
AB126194.2 AATCTTGCTT TCTTCACACT TCCAACCCAA TTTGGAACCT ATGAGTGCAA ACTGCATCTT 720
AY880247.1 AATCTTGCTT TCTTCACACT TCCAACCCAG TTTGGAACCT ATGAGTGCAA ACTGCATCTT 720
Consensus AATCTTGCTT TCTTCACACT TCCAACCCAA TTTGGAACCT ATGAGTGCAA ACTGCATCTT
Conservation 100% 0%
740 760 780
NC_006310.2 GTGGCTTCTT GCTATTTCAT CTATGATAGT AAAGAAGTGT ACAATAAAAG AGGATGTGAC 780
AB126194.2 GTGGCTTCTT GCTATTTCAT CTATGATAGT AAAGAAGTGT ACAATAAAAG AGGATGTGAC 780
AY880247.1 GTGGCTTCTT GCTATTTCAT CTATGATAGT AAAGAAGTGT ACAATAAAAG AGGATGTGAC 780
Consensus GTGGCTTCTT GCTATTTCAT CTATGATAGT AAAGAAGTGT ACAATAAAAG AGGATGTGAC
Conservation 100% 0%
800 820 840
NC_006310.2 AACTACTTTC AAGTGATCTA TGATTCATCT GGAAAAGTTG TTGGAGGACT AGATAACAGG 840
AB126194.2 AACTACTTTC AAGTGATCTA TGATTCATCT GGAAAAGTTG TTGGAGGACT AGATAACAGG 840
AY880247.1 AACTACTTTC AAGTGATCTA TGATTCATCT GGAAAAGTCG TTGGAGGACT AGATAACAGG 840
Consensus AACTACTTTC AAGTGATCTA TGATTCATCT GGAAAAGTTG TTGGAGGACT AGATAACAGG
Conservation 100% 0%
860 880 900
NC_006310.2 GTATCACCTT ACACAGGGAA TTCTGGAGAC ACCCCAACAA TGCAATGTGA CATGCTCCAG 900
AB126194.2 GTATCACCTT ACACAGGGAA TTCTGGAGAC ACCCCAACAA TGCAATGTGA CATGCTCCAG 900
AY880247.1 GTATCACCTT ACACAGGGAA TTCTGGAGAC ACCCCAACAA TGCAATGTGA CATGCTCCAG 900
Consensus GTATCACCTT ACACAGGGAA TTCTGGAGAC ACCCCAACAA TGCAATGTGA CATGCTCCAG
Conservation 100% 0%
920 940 960
NC_006310.2 CTGAAACCTG GAAGATATTC AGTAAGAAGC TCTCCAAGAT TCCTTTTAAT GCCCGAAAGA 960
AB126194.2 CTGAAACCTG GAAGATATTC AGTAAGAAGC TCTCCAAGAT TCCTTTTAAT GCCCGAAAGA 960
AY880247.1 CTGAAACCTG GAAGATATTC AGTAAGAAGC TCTCCAAGGT TCCTTTTAAT GCCTGAAAGA 960
Consensus CTGAAACCTG GAAGATATTC AGTAAGAAGC TCTCCAAGAT TCCTTTTAAT GCCCGAAAGA
Conservation 100% 0%
980 1,000 1,020
NC_006310.2 AGTTATTGCT TTGACATGAA AGAAAAAGGA CCAGTCACTG CTGTCCAATC CATTTGGGGA 1020
AB126194.2 AGTTATTGCT TTGACATGAA AGAAAAAGGA CCAGTCACTG CTGTCCAATC CATTTGGGGA 1020
AY880247.1 AGTTATTGCT TTGACATGAA AGAAAAAGGA CCAGTCACTG CTGTCCAATC CATTTGGGGA 1020
Consensus AGTTATTGCT TTGACATGAA AGAAAAAGGA CCAGTCACTG CTGTCCAATC CATTTGGGGA
Conservation 100% 0%
1,040 1,060 1,080
NC_006310.2 AAAGGCAGAG AATCTGACCA TGCAGTGGAT CAAGCTTGCT TGAGCACTCC AGGGTGCATG 1080
AB126194.2 AAAGGCAGAG AATCTGACCA TGCAGTGGAT CAAGCTTGCT TGAGCACTCC AGGGTGCATG 1080
AY880247.1 AAAGGCAGAG AATCTGACTA TGCAGTGGAT CAAGCTTGCT TGAGCACTCC AGGGTGCATG 1080
Consensus AAAGGCAGAG AATCTGACCA TGCAGTGGAT CAAGCTTGCT TGAGCACTCC AGGGTGCATG
Conservation 100% 0%
1,100 1,120 1,140
NC_006310.2 TTGATCCAAA AGCAAAAGCC ATACATTGGA GAAGCTGATG ATCACCATGG AGATCAAGAA 1140
AB126194.2 TTGATCCAAA AGCAAAAGCC ATACATTGGA GAAGCTGATG ATCACCATGG AGATCAAGAA 1140
AY880247.1 TTGATCCAAA AGCAAAAGCC ATACATTGGA GAAGCTGATG ATCACCATGG AGATCAAGAA 1140
Consensus TTGATCCAAA AGCAAAAGCC ATACATTGGA GAAGCTGATG ATCACCATGG AGATCAAGAA
Conservation 100% 0%
1,160 1,180 1,200
NC_006310.2 ATGAGGGAGT TGCTGTCAGG ACTGGACTAT GAAGCTAGAT GCATATCACA ATCAGGGTGG 1200
AB126194.2 ATGAGGGAGT TGCTGTCAGG ACTGGACTAT GAAGCTAGAT GCATATCACA ATCAGGGTGG 1200
AY880247.1 ATGAGGGAGT TGCTGTCAGG ACTGGACTAT GAAGCTAGAT GCATATCACA ATCAGGGTGG 1200
Consensus ATGAGGGAGT TGCTGTCAGG ACTGGACTAT GAAGCTAGAT GCATATCACA ATCAGGGTGG
Conservation 100% 0%

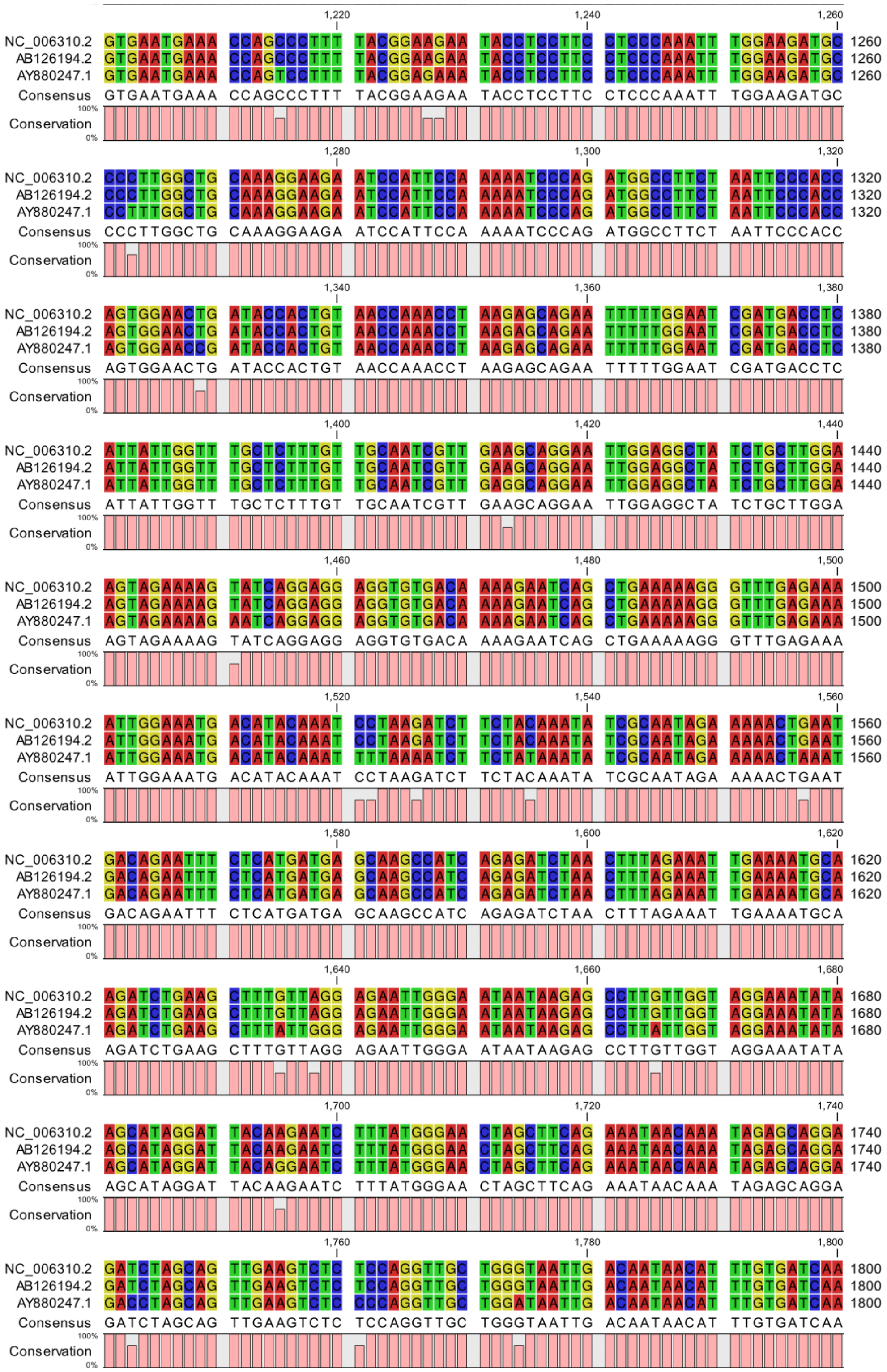
1,220 1,240 1,260
NC_006310.2 GTGAATGAAA CCAGCCCTTT TACGGAAGAA TACCTCCTTC CTCCCAAATT TGGAAGATGC 1260
AB126194.2 GTGAATGAAA CCAGCCCTTT TACGGAAGAA TACCTCCTTC CTCCCAAATT TGGAAGATGC 1260
AY880247.1 GTGAATGAAA CCAGTCCTTT TACGGAGAAA TACCTCCTTC CTCCCAAATT TGGAAGATGC 1260
Consensus GTGAATGAAA CCAGCCCTTT TACGGAAGAA TACCTCCTTC CTCCCAAATT TGGAAGATGC
100% Conservation 0%
1,280 1,300 1,320
NC_006310.2 CCCTTGGCTG CAAAGGAAGA ATCCATTCCA AAAATCCCAG ATGGCCTTCT AATTCCCACC 1320
AB126194.2 CCCTTGGCTG CAAAGGAAGA ATCCATTCCA AAAATCCCAG ATGGCCTTCT AATTCCCACC 1320
AY880247.1 CCTTTGGCTG CAAAGGAAGA ATCCATTCCA AAAATCCCAG ATGGCCTTCT AATTCCCACC 1320
Consensus CCCTTGGCTG CAAAGGAAGA ATCCATTCCA AAAATCCCAG ATGGCCTTCT AATTCCCACC
100% Conservation 0%
1,340 1,360 1,380
NC_006310.2 AGTGGAACTG ATACCACTGT AACCAAACCT AAGAGCAGAA TTTTTGGAAT CGATGACCTC 1380
AB126194.2 AGTGGAACTG ATACCACTGT AACCAAACCT AAGAGCAGAA TTTTTGGAAT CGATGACCTC 1380
AY880247.1 AGTGGAACCG ATACCACTGT AACCAAACCT AAGAGCAGAA TTTTTGGAAT CGATGACCTC 1380
Consensus AGTGGAACTG ATACCACTGT AACCAAACCT AAGAGCAGAA TTTTTGGAAT CGATGACCTC
100% Conservation 0%
1,400 1,420 1,440
NC_006310.2 ATTATTGGTT TGCTCTTTGT TGCAATCGTT GAAGCAGGAA TTGGAGGCTA TCTGCTTGGA 1440
AB126194.2 ATTATTGGTT TGCTCTTTGT TGCAATCGTT GAAGCAGGAA TTGGAGGCTA TCTGCTTGGA 1440
AY880247.1 ATTATTGGTT TGCTCTTTGT TGCAATCGTT GAGGCAGGAA TTGGAGGCTA TCTGCTTGGA 1440
Consensus ATTATTGGTT TGCTCTTTGT TGCAATCGTT GAAGCAGGAA TTGGAGGCTA TCTGCTTGGA
100% Conservation 0%
1,460 1,480 1,500
NC_006310.2 AGTAGAAAAG TATCAGGAGG AGGTGTGACA AAAGAATCAG CTGAAAAAGG GTTTGAGAAA 1500
AB126194.2 AGTAGAAAAG TATCAGGAGG AGGTGTGACA AAAGAATCAG CTGAAAAAGG GTTTGAGAAA 1500
AY880247.1 AGTAGAAAAG AATCAGGAGG AGGTGTGACA AAAGAATCAG CTGAAAAAGG GTTTGAGAAA 1500
Consensus AGTAGAAAAG TATCAGGAGG AGGTGTGACA AAAGAATCAG CTGAAAAAGG GTTTGAGAAA
100% Conservation 0%
1,520 1,540 1,560
NC_006310.2 ATTGGAAATG ACATACAAAT CCTAAGATCT TCTACAAATA TCGCAATAGA AAAACTGAAT 1560
AB126194.2 ATTGGAAATG ACATACAAAT CCTAAGATCT TCTACAAATA TCGCAATAGA AAAACTGAAT 1560
AY880247.1 ATTGGAAATG ACATACAAAT TTTAAAATCT TCTATAAATA TCGCAATAGA AAAACTAAAT 1560
Consensus ATTGGAAATG ACATACAAAT CCTAAGATCT TCTACAAATA TCGCAATAGA AAAACTGAAT
100% Conservation 0%
1,580 1,600 1,620
NC_006310.2 GACAGAATTT CTCATGATGA GCAAGCCATC AGAGATCTAA CTTTAGAAAT TGAAAATGCA 1620
AB126194.2 GACAGAATTT CTCATGATGA GCAAGCCATC AGAGATCTAA CTTTAGAAAT TGAAAATGCA 1620
AY880247.1 GACAGAATTT CTCATGATGA GCAAGCCATC AGAGATCTAA CTTTAGAAAT TGAAAATGCA 1620
Consensus GACAGAATTT CTCATGATGA GCAAGCCATC AGAGATCTAA CTTTAGAAAT TGAAAATGCA
100% Conservation 0%
1,640 1,660 1,680
NC_006310.2 AGATCTGAAG CTTTGTTAGG AGAATTGGGA ATAATAAGAG CCTTGTTGGT AGGAAATATA 1680
AB126194.2 AGATCTGAAG CTTTGTTAGG AGAATTGGGA ATAATAAGAG CCTTGTTGGT AGGAAATATA 1680
AY880247.1 AGATCTGAAG CTTTATTGGG AGAATTGGGA ATAATAAGAG CCTTATTGGT AGGAAATATA 1680
Consensus AGATCTGAAG CTTTGTTAGG AGAATTGGGA ATAATAAGAG CCTTGTTGGT AGGAAATATA
100% Conservation 0%
1,700 1,720 1,740
NC_006310.2 AGCATAGGAT TACAAGAATC TTTATGGGAA CTAGCTTCAG AAATAACAAA TAGAGCAGGA 1740
AB126194.2 AGCATAGGAT TACAAGAATC TTTATGGGAA CTAGCTTCAG AAATAACAAA TAGAGCAGGA 1740
AY880247.1 AGCATAGGAT TACAGGAATC TTTATGGGAA CTAGCTTCAG AAATAACAAA TAGAGCAGGA 1740
Consensus AGCATAGGAT TACAAGAATC TTTATGGGAA CTAGCTTCAG AAATAACAAA TAGAGCAGGA
100% Conservation 0%
1,760 1,780 1,800
NC_006310.2 GATCTAGCAG TTGAAGTCTC TCCAGGTTGC TGGGTAATTG ACAATAACAT TTGTGATCAA 1800
AB126194.2 GATCTAGCAG TTGAAGTCTC TCCAGGTTGC TGGGTAATTG ACAATAACAT TTGTGATCAA 1800
AY880247.1 GACCTAGCAG TTGAAGTCTC CCCAGGTTGC TGGATAATTG ACAATAACAT TTGTGATCAA 1800
Consensus GATCTAGCAG TTGAAGTCTC TCCAGGTTGC TGGGTAATTG ACAATAACAT TTGTGATCAA
100% Conservation 0%

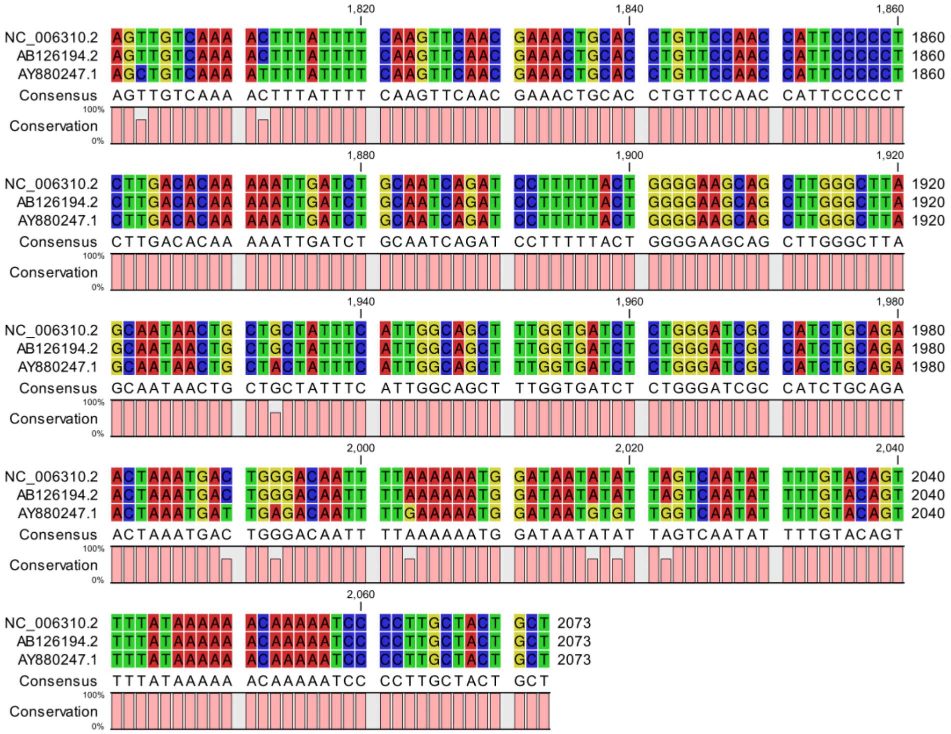

Figure S1. Alignment of selected sequences

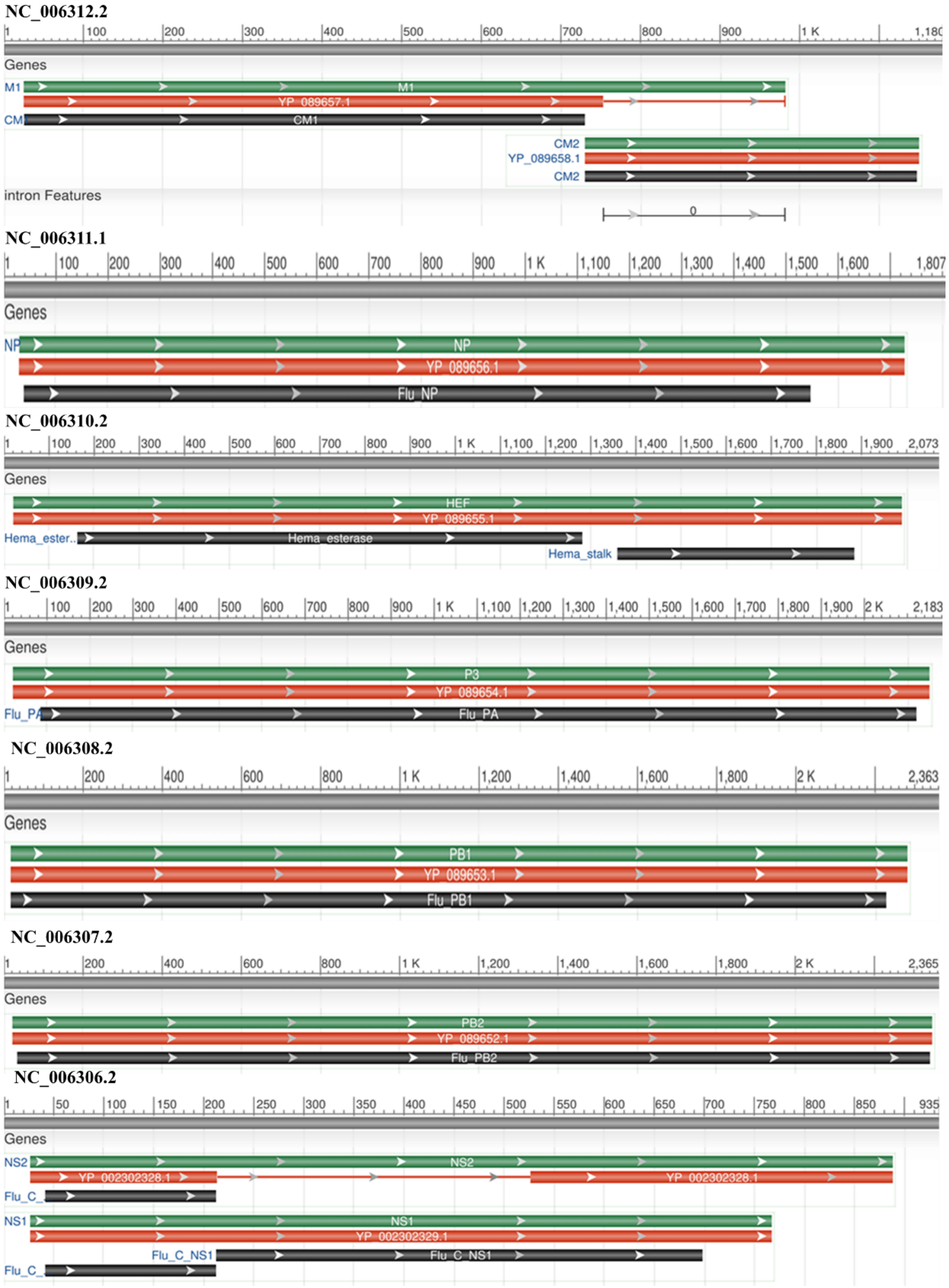

Figure S2. Genomic regions

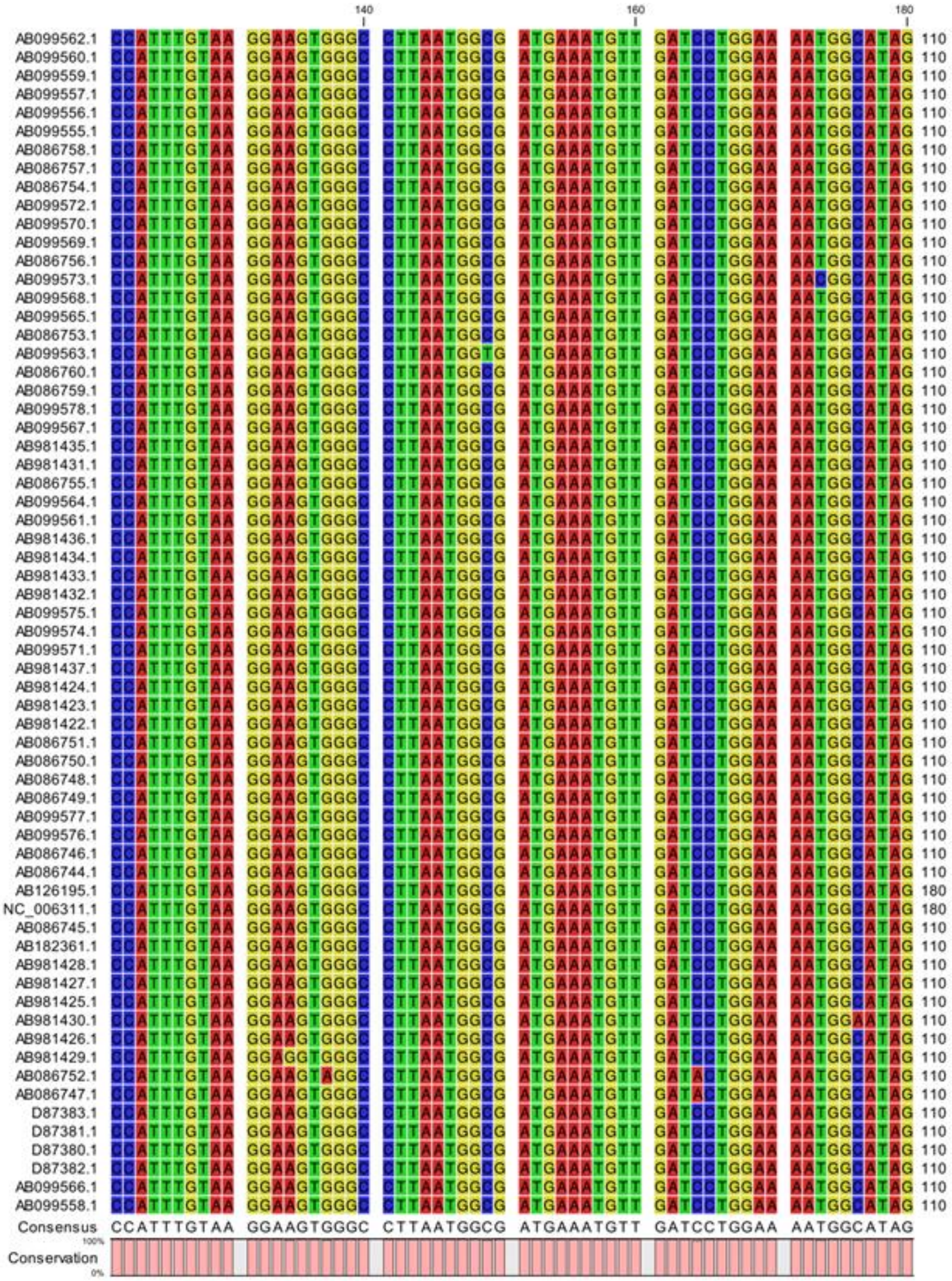

Figure S3. Conservation pattern of nucleotides sequences

```
                      cov    pid   81      .         1         .         .         .         :         . 160
1 Mississippi/80   100.0% 100.0%      -------AGTCAAATCAACAA------------ATTTAATGGC----------ATTTGTA-GCCAC---------AAAAA
2 TAYLOR/1233/47   100.0%  98.7%      -------AGTCAAATCAACAA------------ATTTAATGGC----------ATTTGTA-GCCAC---------AAAAA
3 Yamagata/26/81    99.0%  27.5%      CCTTCAAAAGCAAGTGAACAGTAGCTTCAGCCTACACAATGGCTTTGGAGGAAATTTGTATGCCACAGAAGAAAAAAGAA
4 Sao-Paulo/378/82  99.0%  28.3%      CCTTCAAAAGCAAGTGAACAGTAGCTTCAGCCTACACAATGGCTTCGGAGGAAATTTGTATGCCACAGAAGAAAAAAGAA
5 Arbor/1/50        99.0%  27.1%      CCTTCAAAAGCAAGTGAACAGTAGCTTCAGCCTACACAATGGCTTCGGAGGAAATTTGTATGCCACGGAAGAAAAAAGAA
6 Aichi/1/81        99.0%  28.0%      CCTTCAAAAGCAAGTGAACAGTAGCTTCAGCCTACACAATGGCTTCGGAGGAAATTTGTATGCCACAGAAGAAAAAAGAA
7 JHB/1/66          99.0%  27.1%      CCTTCAAAAGCAAGTGAACAGTAGCTTCAGCCTACACAATGGCTTCGGAGGAAATTTGTATGCCACAGAAGAAAAAAGAA
  consensus/100%                      .......AusCAAuTsAACAu............AsssAATGGC..........ATTTGTA.GCCAC.........AAuAA
  consensus/90%                       .......AusCAAuTsAACAu............AsssAATGGC..........ATTTGTA.GCCAC.........AAuAA
  consensus/80%                       .......AusCAAuTsAACAu............AsssAATGGC..........ATTTGTA.GCCAC.........AAuAA
  consensus/70%                       CCTTCAAAAGCAAGTGAACAGTAGCTTCAGCCTACACAATGGCTTsGGAGGAAATTTGTATGCCACuGAAGAAAAAAGAA

                      cov    pid   161     .         .         .         2         .         .         .         . 240
1 Mississippi/80   100.0% 100.0%      TGTTAGAG-------AGACAAGA-----------------AGATTTAGACACATGCACT-------GAAATGCAAGTA--
2 TAYLOR/1233/47   100.0%  98.7%      TGTTAGAG-------AGACAAGA-----------------AGATTTAGACACATGCACT-------GAAATGCAAGTA--
3 Yamagata/26/81    99.0%  27.5%      TGTTTGAGCTTGTTAAGCCCAAAGCTGGAGCCTCTGTCTTGAATCAAAGTACATGGATCGGCTTTGGAGATTCAAGAACT
4 Sao-Paulo/378/82  99.0%  28.3%      TGTTTGAGCTTGTTAAGCCCAAAGCTGGAGCCTCTGTCTTGAATCAAAGTACATGGATTGGCTTTGGAGATTCAAGGACT
5 Arbor/1/50        99.0%  27.1%      TGTTTGAGCTTGTTAAGCCCAAAGCTGGAGCCTCTGTCTTGAATCAAAGTACATGGATTGGCTTTGGAGATTCAAGGACT
6 Aichi/1/81        99.0%  28.0%      TGTTTGAGCTTGTTAAGCCTAAAGCTGGAGCCTCTGTCTTGAATCAAAGTACATGGATTGGCTTTGGAGATTCAAGGACT
7 JHB/1/66          99.0%  27.1%      TGTTTGAGCTTGTTAAGCCCAAAGCTGGAGCCTCTGTCTTGAATCAAAGTACATGGATTGGCTTTGGAGATTCAAGGACT
  consensus/100%                      TGTTsGAG.......AGsCsAuA.................uuATssAuusACATGsAss.......GAuATsCAAGsA..
  consensus/90%                       TGTTsGAG.......AGsCsAuA.................uuATssAuusACATGsAss.......GAuATsCAAGsA..
  consensus/80%                       TGTTsGAG.......AGsCsAuA.................uuATssAuusACATGsAsT.......GAuATsCAAGsA..
  consensus/70%                       TGTTTGAGCTTGTTAAGCCsAAAGCTGGAGCCTCTGTCTTGAATCAAAGTACATGGATTGGCTTTGGAGATTCAAGuACT

                      cov    pid   241     :         .         .         .         .         3         .         . 320
1 Mississippi/80   100.0% 100.0%      GAAAAAATGAAAACG------------------------TCAACAAAAGC------CAGATT------------------
2 TAYLOR/1233/47   100.0%  98.7%      GAAAAAATGAAAACG------------------------TCAACAAAAGC------TAGGTT------------------
3 Yamagata/26/81    99.0%  27.5%      GACCAAAGCAATTCAGCTTTTCCTAGGTCTGCGGATGTTTCAGCAAAAACTGCTGATAAGTTTCGTTCTTTATCTGGTGG
4 Sao-Paulo/378/82  99.0%  28.3%      GACAAAACCAATTCAGCTTTTCCTAGGTCTGCTGATGTTTCAGCAAAGACTGCTGATAAATTTCGTTCTTTGTCTGGTGG
5 Arbor/1/50        99.0%  27.1%      GACAAAAGCAATTCAGCTTTTCCTAGGTCTGCGGATGTTTCAGCAAAAACTGCTGATAAGTTTCGTTCTTTGTCTGGTGG
6 Aichi/1/81        99.0%  28.0%      GACAAAAGCAATTCAGCTTTTCCTAGGTCTGCTGATGTTTCAGCAAAAACTGCTAATAAGTTTCGTTCTTTGTCTGGTGG
7 JHB/1/66          99.0%  27.1%      GACAAAAGCAATTCAGCTTTTCCTAGGTCTGCTGATGTTTCAGCAAAAACTGCTGATAAGTTTCGTTCTTTGTCTGGTGG
  consensus/100%                      GAssAAAssAAssCu........................TCAuCAAAuuC......sAuuTT..................
  consensus/90%                       GAssAAAssAAssCu........................TCAuCAAAuuC......sAuuTT..................
  consensus/80%                       GAsAAAAssAAssCu........................TCAuCAAAAuC......TAuuTT..................
  consensus/70%                       GACAAAAsCAATTCAGCTTTTCCTAGGTCTGCsGATGTTTCAGCAAAAACTGCTuATAAGTTTCGTTCTTTuTCTGGTGG

                      cov    pid   321     .         .         :         .         .         .         .       4 400
1 Mississippi/80   100.0% 100.0%      ---------------------------------GAAAACAGAATCCTCTTTTGCACCTAGAACATGGGAA----------
2 TAYLOR/1233/47   100.0%  98.7%      ---------------------------------GAGAACAGAATCTTCTTTTGCACCTAGAACATGGGAA----------
3 Yamagata/26/81    99.0%  27.5%      ATCCTTGATGTTGAGTATGTTTGGCCCACCTGGGAAGGTAGATTACCTTT----ACCAAGGATGTGGAAAGCATAAAGTT
4 Sao-Paulo/378/82  99.0%  28.3%      ATCCTTGATGCTGAGTATGTTTGGCCCACCTGGGAAGGTAGATTACCTTT----ACCAAGGATGTGGAAAACATAAGGTT
5 Arbor/1/50        99.0%  27.1%      ATCCTTGATGTTGAGTATGTTTGGACCACCTGGGAAGGTAGACTACCTTT----ACCAAGGATGTGGAAAACATAAGGTT
6 Aichi/1/81        99.0%  28.0%      ATCCTTAATGTTGAGTATGTTTGGCCCACCTGGGAAGGTAGACTACCTTT----ACCAAGGATGTGGAAAACATAAAGTT
7 JHB/1/66          99.0%  27.1%      ATCCTTAATGTTGAGTATGTTTGGCCCACCTGGGAAGGTAGACTACCTTT----ACCAAGGATGTGGAAAACATAAAGTT
  consensus/100%                      .................................GAuuusAGAsTssssTT....ACCsAGuAsuTGGuAA..........
  consensus/90%                       .................................GAuuusAGAsTssssTT....ACCsAGuAsuTGGuAA..........
  consensus/80%                       .................................GAAuusAGAsTsCssTT....ACCsAGuAsuTGGuAA..........
  consensus/70%                       ATCCTTuATGsTGAGTATGTTTGGsCCACCTGGGAAGGTAGAsTACCTTT....ACCAAGGATGTGGAAAuCATAAuGTT

                      cov    pid   401     .         .         .         .         :         .         .         . 480
1 Mississippi/80   100.0% 100.0%      -------------------------------------------GATGCAATAAAAGATGG-------------TGAGCTT
2 TAYLOR/1233/47   100.0%  98.7%      -------------------------------------------GATGCAATAAAAGATGG-------------TGAGCTT
3 Yamagata/26/81    99.0%  27.5%      TTTTATGAAGGAGTCAACTGGAGTCCACATGCTGCTATAGATTGTTACAGAAAAAATTGGACTGACATCAAACTAAATTT
4 Sao-Paulo/378/82  99.0%  28.3%      TTTTATGAAGGAGTTAACTGGAGTCCACATGCTGCTATAGATTGTTACAGTAAAAATTGGACTGATATCAAACTGAATTT
5 Arbor/1/50        99.0%  27.1%      TTTTATGAAGGAGTTAACTGGAGTCCACATGCTGCTATAAATTGTTACAGAAAAAATTGGACTGATATCAAACTGAATTT
6 Aichi/1/81        99.0%  28.0%      TTTTATGAAGGAGTTAACTGGAGCCCACATGCTGCTATAAATTGCTACAGAAAAAATTGGACTGACATCAAACTGAATTT
7 JHB/1/66          99.0%  27.1%      TTTTATGAAGGAGTTAACTGGAGTCCACATGCTGCTATAAATTGTTACAGAAAAAATTGGACTGATATCAAACTGAATTT
  consensus/100%                      ...........................................GsTuCAusAAAAusTGG.............TuAusTT
  consensus/90%                       ...........................................GsTuCAusAAAAusTGG.............TuAusTT
  consensus/80%                       ...........................................GsTuCAusAAAAusTGG.............TGAusTT
  consensus/70%                       TTTTATGAAGGAGTsAACTGGAGsCCACATGCTGCTATAuATTGsTACAGsAAAAATTGGACTGAsATCAAACTGAATTT

                      cov    pid   481     .         5         .         .         .         .         :         . 560
1 Mississippi/80   100.0% 100.0%      CTA-------------------------------------------------------TTCAACGGGACGATTC------
2 TAYLOR/1233/47   100.0%  98.7%      CTA-------------------------------------------------------TTCAACGGGACGATTC------
3 Yamagata/26/81    99.0%  27.5%      CCAGAAAAGCATTTATGAATTGGCTTCACAATCACATTGCATGAGCTTGGTGAATGCCTTGGACAAAACTATTCCTTTAC
4 Sao-Paulo/378/82  99.0%  28.3%      CCAGAAAAACATTTATGAATTGGCTTCAAAATCACATTGCATGAGCTTGGTGAATGCCTTGGACAAAACTATTCCTTTAC
5 Arbor/1/50        99.0%  27.1%      CCAGAAAAACATTTATGAACTGGCTTCACAATCACATTGCATGAGCTTGGTGAATGCCTTGGACAAAACTATTCCTTTAC
6 Aichi/1/81        99.0%  28.0%      CCAGAAAAACATTTATGAATTGGCTTCACAATCACATTGCATGAGCTTGGTGAATGCCTTGGACAAAACTATTCCTTTAC
7 JHB/1/66          99.0%  27.1%      CCAGAAAAACATTTATGAATTGGCTTCACAATCACATTGCATGAGCTTGGTGAATGCCTTGGACAAAACTATTCCTTTAC
  consensus/100%                      CsA.......................................................TTsuACuuuACsATTC......
  consensus/90%                       CsA.......................................................TTsuACuuuACsATTC......
  consensus/80%                       CsA.......................................................TTsuACuuuACsATTC......
  consensus/70%                       CCAGAAAAuCATTTATGAAsTGGCTTCAsAATCACATTGCATGAGCTTGGTGAATGCCTTGGACAAAACTATTCCTTTAC
```

```
                          cov     pid   561         .         .         .         6         .         .         .         . 640
1 Mississippi/80      100.0% 100.0%      --------------------------TGCAAGCAGAGTCTCCTACAATGACGCCAGCGTC--------------------
2 TAYLOR/1233/47      100.0%  98.7%      --------------------------TGCAAGCAGAGTCCCCTACAATGACGCCAGCGTC--------------------
3 Yamagata/26/81       99.0%  27.5%      AAGTGACTAAAGGAGTTGCAAAAAATTGCAACAACAGCTTCTTAAAA--AATCCAGCATTGTACACACAAGAAGTCAAAC
4 Sao-Paulo/378/82     99.0%  28.3%      ATGCGACTGCTGGGACTGCAAAAAATTGCAACAACAGTTTCTTAAAA--AATCCAGCATTGTACACACAAGAAGTTAAAC
5 Arbor/1/50           99.0%  27.1%      AAGCGACTGCAGGGGTTGCAAAAAATTGCAACAACAGCTTCTTAAAA--AATCCAGCATTGTACACACAAGAAGTCAATC
6 Aichi/1/81           99.0%  28.0%      AAGTGACTGCTGGGACTGCAGGAAATTGCAACAACAGCTTCTTAAAA--AATCCAGCATTGTACACACAAGAAGTCAAGC
7 JHB/1/66             99.0%  27.1%      AAGTGACTGCTGGGACTGCAGGAAATTGCAACAACAGCTTCTTAAAA--AATCCAGCATTGTACACACAAGAAGTCAAGC
  consensus/100%                         ..........................TGCAAssAsAGsssCsTAsAA..AssCCAGCuTs....................
  consensus/90%                          ..........................TGCAAssAsAGsssCsTAsAA..AssCCAGCuTs....................
  consensus/80%                          ..........................TGCAAssAsAGssTCsTAsAA..AssCCAGCuTs....................
  consensus/70%                          AsGsGACTussGGuusTGCAuuAAATTGCAACAACAGsTTCTTAAAA..AATCCAGCATTGTACACACAAGAAGTsAAsC
```

```
                          cov     pid   641         :         .         .         .         .         7         .         . 720
1 Mississippi/80      100.0% 100.0%      ------TGTAGAAATGAAGGGGAAGAAACTTCCTATTGATTTTGCTCCAAG--CAACATAGCACCAATTGG-GCAAAAT-
2 TAYLOR/1233/47      100.0%  98.7%      ------CGTAGAAATGAAGGGAAAGAAATTTCCTATTGATTTTGCTCCAAG--CAACATAGCACCAATTGG-GCAAAAT-
3 Yamagata/26/81       99.0%  27.5%      CTTTAGAGCAAATATGTGGAGAAGAAAATCTTGCTTTCTTCACACTTCCAACCCAATTTGGAACCTATGAGTGCAAACTG
4 Sao-Paulo/378/82     99.0%  28.3%      CTTCAGAAGAAAAATGTGGAAAGAGAATCTTGCTTTCTTCACACTTCCAACCCAATTTGGAACCTATGAGTGCAAACTG
5 Arbor/1/50           99.0%  27.1%      CTTCAGTAGAAAAATGTGGAAAGAAAATCTTGCTTTCTTCACACTTCCAACCCAATTTGGAACCTATGAGTGCAAACTG
6 Aichi/1/81           99.0%  28.0%      CTTCAGAAAACAAATGTGGGAAGAAAATCTTGCTTTCTTCACACTTCCAACCCAATTTGGAACCTATGAGTGCAAACTG
7 JHB/1/66             99.0%  27.1%      CTTCAGAAAACAAATGTGGAAAGAAAATCTTGCTTTCTTCACACTTCCAACCCAGTTTGGAACCTATGAGTGCAAACTG
  consensus/100%                         ......susAsAsATGsuGuuuAuuuAAssTssssTTssTsssuCTsCsAu..CAussTuGsACCsATsuG.GCAAAsT.
  consensus/90%                          ......susAsAsATGsuGuuuAuuuAAssTssssTTssTsssuCTsCsAu..CAussTuGsACCsATsuG.GCAAAsT.
  consensus/80%                          ......susAsAAATGsuGGuAAuuAAATsTssssTTssTsssuCTsCsAu..CAAssTuGsACCsATsuG.GCAAAsT.
  consensus/70%                          CTTsAGsusAuAAATGTGGGuAAGAAAATCTTGCTTTCTTCACACTTCCAACCCAATTTGGAACCTATGAGTGCAAACTG
```

```
                          cov     pid   721         .         .         :         .         .         .         .         8 800
1 Mississippi/80      100.0% 100.0%      -----------------CCAATATATTTATCAC--------------------------------CATGTATTCCTAA--
2 TAYLOR/1233/47      100.0%  98.7%      -----------------CCAATATATCTGTCAC--------------------------------CATGTATTCCTAA--
3 Yamagata/26/81       99.0%  27.5%      CATCTTGTGGCTTCTTGCTATTTCATCTATGATAGCAAAGAAGTGTACAATAAAAGAGGATGTGGCAACTACTTTCAAGT
4 Sao-Paulo/378/82     99.0%  28.3%      CATCTTGTGGCTTCTTGCTATTTCATTTATGATAGTAAAGAAGTGTACAATAAAAGAGGGTGTGACAACTACTTTCAAGT
5 Arbor/1/50           99.0%  27.1%      CATCTTGTGGCTTCTTGCTATTTCATCTATGATAGTAAAGAAGTGTACAATAAAAGAGGATGTGACAACTACTTTCAAGT
6 Aichi/1/81           99.0%  28.0%      CATCTTGTGGCTTCTTGCTATTTCATCTATGACAGTAAAGAAGTGTACAATAAAAGAGGATGTGGCAACTACTTTCAAGT
7 JHB/1/66             99.0%  27.1%      CATCTTGTGGCTTCTTGCTATTTCATCTATGATAGTAAAGAAGTGTACAATAAAAGAGGATGTGACAACTACTTTCAAGT
  consensus/100%                         .................CsAsTssATsTuTsAs................................CAssTAsTsssAA..
  consensus/90%                          .................CsAsTssATsTuTsAs................................CAssTAsTsssAA..
  consensus/80%                          .................CsAsTssATsTATsAs................................CAssTAsTsssAA..
  consensus/70%                          CATCTTGTGGCTTCTTGCTATTTCATCTATGAsAGsAAAGAAGTGTACAATAAAAGAGGuTGTGuCAACTACTTTCAAGT
```

```
                          cov     pid   801         .         .         .         .         :         .         .         . 880
1 Mississippi/80      100.0% 100.0%      ---CTTTGA------TGGAAACGTC-TGGGAAG------CAACGATGTATCATC-----------ATCGTGGAGCAACTT
2 TAYLOR/1233/47      100.0%  98.7%      ---CTTTGA------TGGAAACGTC-TGGGAAG------CAACGATGTATCATC-----------ATCGTGGAGCAACTT
3 Yamagata/26/81       99.0%  27.5%      GATCTATGATTCATCTGGAAAAGTTGTTGGAGGGCTAGATAACAGGGTATCACCTTACACAGGGAATTCTGGAGACACTC
4 Sao-Paulo/378/82     99.0%  28.3%      GATCTATGATTCATCTGGAAAAGTTGTTGGAGGACTAGATAACAGGGTATCACCTTACACAGGGAATTCTGGAGACACCC
5 Arbor/1/50           99.0%  27.1%      GATCTATGATTCATCTGGAAAAGTTGTTGGAGGACTAGATAACAGGGTATCACCTTACACAGGGAATTCTGGAGACACCC
6 Aichi/1/81           99.0%  28.0%      GATCTATGATTCATCTGGAAAAGTTGTTGGAGGACTAGATAACAGGGTATCACCTTACACAGGGAATTCTGGGGACACCC
7 JHB/1/66             99.0%  27.1%      GATCTATGATTCATCTGGAAAAGTCGTTGGAGGACTAGATAACAGGGTATCACCTTACACAGGGAATTCTGGAGACACCC
  consensus/100%                         ...CTsTGA......TGGAAAsGTs.TsGGAuG......sAACuusGTATCAsC...........ATssTGGuGssACss
  consensus/90%                          ...CTsTGA......TGGAAAsGTs.TsGGAuG......sAACuusGTATCAsC...........ATssTGGuGssACss
  consensus/80%                          ...CTsTGA......TGGAAAsGTs.TsGGAuG......sAACuusGTATCAsC...........ATssTGGAGssACss
  consensus/70%                          GATCTATGATTCATCTGGAAAAGTsGTTGGAGGuCTAGATAACAGGGTATCACCTTACACAGGGAATTCTGGAGACACsC
```

```
                          cov     pid   881         .         9         .         .         .         .         :         . 960
1 Mississippi/80      100.0% 100.0%      TGACAAAGACAATG-----------AATTGCAAC----------------------------------------------
2 TAYLOR/1233/47      100.0%  98.7%      TGACAAAGACAATG-----------AATTGCAAC----------------------------------------------
3 Yamagata/26/81       99.0%  27.5%      CAACAATG-CAATGTGACATGCTCCAGCTGAAACCTGGAAGATATTCAGTAAGAAGCTCTCCAAGATTCCTTTTAATGCC
4 Sao-Paulo/378/82     99.0%  28.3%      CAACAATG-CAATGTGACATGATCCAGCTGAAACCTGGAAGATATTCAGTAAGAAGCTCTCCAAGATTCCTTTTAATGCC
5 Arbor/1/50           99.0%  27.1%      CAACAATG-CAATGTGACATGCTCCAGCTGAAACCTGGAAGATATTCAGTAAGAAGCTCTCCAAGATTCCTTTTAATGCC
6 Aichi/1/81           99.0%  28.0%      CAACAATG-CAATGTGACATGCTCCAGCTGAAACCTGGAAGATATTCAGTAAGAAGCTCTCCAAGATTCCTTTTAATGCC
7 JHB/1/66             99.0%  27.1%      CAACAATG-CAATGTGACATGCTCCAGCTGAAACCTGGAAGATATTCAGTAAGAAGCTCTCCAAGGTTCCTTTTAATGCC
  consensus/100%                         suACAAsG.CAATG...........AusTGsAAC..............................................
  consensus/90%                          suACAAsG.CAATG...........AusTGsAAC..............................................
  consensus/80%                          suACAAsG.CAATG...........AusTGsAAC..............................................
  consensus/70%                          CAACAATG.CAATGTGACATGsTCCAGCTGAAACCTGGAAGATATTCAGTAAGAAGCTCTCCAAGuTTCCTTTTAATGCC
```

```
                          cov     pid   961         .         .         .         0         .         .         .         . 1040
1 Mississippi/80      100.0% 100.0%      -------------TGTTTT-----------CAAAGAACAATTTGGTGCCATCCAAATCCTTCACGTATGAGATTGAG---
2 TAYLOR/1233/47      100.0%  98.7%      -------------TGTTTT-----------CAAAGAACAATTTGGTGCCATCCAAATCCTTCACGTATGAGATTGAG---
3 Yamagata/26/81       99.0%  27.5%      TGAAAGGAGTTATTGCTTTGACATGAAAGAAAAAGGACCAGTCACTGCTGTCCAATCCATCTGGGGAAAGGCAGAAAAT
4 Sao-Paulo/378/82     99.0%  28.3%      TGAGAGAAGTTATTGCTTTGACATGAAAGAAAAAGGACCAGTCACTGCTGTTCAATCCGTTTGGGGAAAGGCAGAAAAT
5 Arbor/1/50           99.0%  27.1%      CGAAAGAAGTTATTGCTTTGACATGAAAGAAAAAGGACCAGTCACTGCTGTCCAATCCATTTGGGGAAAGGCAGAGAAT
6 Aichi/1/81           99.0%  28.0%      TGAAAGAAGTTATTGCTTTGACATGAAAGAAAAAGGACCAGTCACTGCTGTCCAATCCATTTGGGGAAAGGCAGAGAAT
7 JHB/1/66             99.0%  27.1%      TGAAAGAAGTTATTGCTTTGACATGAAAGAAAAAGGACCAGTCACTGCTGTCCAATCCATTTGGGGAAAGGCAGAGAAT
  consensus/100%                         .............TGsTTT...........sAAAGuACsAsTsusTGCsuTsCAAssCsTssusGsAsuAGussGAu...
  consensus/90%                          .............TGsTTT...........sAAAGuACsAsTsusTGCsuTsCAAssCsTssusGsAsuAGussGAu...
  consensus/80%                          .............TGsTTT...........sAAAGuACsAsTsusTGCsuTCCAAssCsTTsusGsAsuAGussGAu...
  consensus/70%                          sGAuAGuAGTTATTGCTTTGACATGAAAGAAAAAGGACCAGTCACTGCTGTCCAATCCuTTTGGGGAAAGGCAGAGAAT
```

```
                         cov     pid 1041        :         .         .         .         .         1         .         . 1120
1 Mississippi/80      100.0% 100.0%  ----CTATGCA----------TTTGTTT----------------------------------------TGTATTGCAGAA
2 TAYLOR/1233/47      100.0%  98.7%  ----CTATGCA----------TTTGTTT----------------------------------------TGTATTGCAGAA
3 Yamagata/26/81       99.0%  27.5%  CTGACTATGCAGTAGATCAGGCTTGCTTAAGCACTCCAGGGTGCATGTTGATCCAAAAGCAAAAGCCATACATTGGAGAG
4 Sao-Paulo/378/82     99.0%  28.3%  CTGACTATGCAGTGGATCAAGCTTGCTTGAGCACTCCAGGGTGCATGTTGATCCAAAAGCAAAAGCCGTACATTGGAGAA
5 Arbor/1/50           99.0%  27.1%  CTGACCATGCAGTGGATCAAGCTTGCTTGAGCACTCCAGGGTGCATGTTGATCCAAAAGCAAAAGCCATACATTGGAGAA
6 Aichi/1/81           99.0%  28.0%  CTGACTATGCAGTGGATCAAGCTTGCTTGAGCACTCCAGGGTGCATGTTGATCCAAAAGCAAAAGCCATACATTGGAGAA
7 JHB/1/66             99.0%  27.1%  CTGACTATGCAGTGGATCAAGCTTGCTTGAGCACTCCAGGGTGCATGTTGATCCAAAAGCAAAAGCCATACATTGGAGAA
  consensus/100%                     ....CsATGCA..........sTTGsTT........................................TusATTGsAGAu
  consensus/90%                      ....CsATGCA..........sTTGsTT........................................TusATTGsAGAu
  consensus/80%                      ....CTATGCA..........sTTGsTT........................................TusATTGsAGAA
  consensus/70%                      CTGACTATGCAGTuGATCAuGCTTGCTTuAGCACTCCAGGGTGCATGTTGATCCAAAAGCAAAAGCCuTACATTGGAGAA
```

```
                         cov     pid 1121        .         .         :         .         .         .         .       2 1200
1 Mississippi/80      100.0% 100.0%  A--------TACTAAGAAGATCTGTGGATA----------CCTCATCGCTAGACAA------------------------
2 TAYLOR/1233/47      100.0%  98.7%  A--------TACTAAGAAGATCTGTGGATA----------CCTCATCGCTAGACAA------------------------
3 Yamagata/26/81       99.0%  27.5%  GCTGATGATCACCATGGAGATCAAGAAATGAGGGAGTTGCTGTCAGGACTGGACTATGAAGCTAGATGCATATCACAATC
4 Sao-Paulo/378/82     99.0%  28.3%  GCTGATGATCACCATGGAGATCAAGAAATGAGGGAGTTGCTGTCAGGACTGGACTATGAAGCTAGATGTGTATCACAATC
5 Arbor/1/50           99.0%  27.1%  GCTGATGATCACCATGGAGATCAAGAAATGAGGGAGTTGCTGTCAGGACTGGACTATGAAGCTAGATGCATATCACAATC
6 Aichi/1/81           99.0%  28.0%  GCTGATGATCACCATGGAGATCAAGAAATGAGGGAGTTGCTGTCAGGACTGGACTATGAAGCTAGATGCATATCACAATC
7 JHB/1/66             99.0%  27.1%  GCTGATGATCACCATGGAGATCAAGAAATGAGGGAGTTGCTGTCAGGACTGGACTATGAAGCTAGATGCATATCACAATC
  consensus/100%                     u........sACsAsGuAGATCsusuuATu..........ssTCAssuCTuGACsA........................
  consensus/90%                      u........sACsAsGuAGATCsusuuATu..........ssTCAssuCTuGACsA........................
  consensus/80%                      u........sACsAsGuAGATCsusuuATu..........ssTCAssuCTuGACsA........................
  consensus/70%                      GCTGATGATCACCATGGAGATCAAGAAATGAGGGAGTTGCTGTCAGGACTGGACTATGAAGCTAGATGsuTATCACAATC
```

```
                         cov     pid 1201        .         .         .         .         :         .         .         . 1280
1 Mississippi/80      100.0% 100.0%  ---GTGGCCGGAATTGAAAC--------------AGGAA-------------------TTAGAAAATG--TTTCAGATGC
2 TAYLOR/1233/47      100.0%  98.7%  ---GTGGCCGGAATTGAAAC--------------AGGAA-------------------TTAGAAAATG--TTTCAGATGC
3 Yamagata/26/81       99.0%  27.5%  AGGGTGGGTGAA--TGAAACCAGTCCTTTTACGGAAGAATACCTCCTTCCTCCCAAATTTGGAAGATGCCCCTTGGCTGC
4 Sao-Paulo/378/82     99.0%  28.3%  AGGGTGGGTGAA--TGAAACCAGCCCTTTTGCGGAGGAATACCTCCTCCCTCCCAAATTTGGAAGATGCCCCTTGGCTGC
5 Arbor/1/50           99.0%  27.1%  AGGGTGGGTGAA--TGAAACCAGCCCTTTTACGGAAGAATACCTCCTTCCTCCCAAATTTGGAAGATGCCCCTTGGCTGC
6 Aichi/1/81           99.0%  28.0%  AGGGTGGGTGAA--CGAAACCAGTCCTTTTACGGAGAAATACCTCCTTCCTCCCAAATTTGGAAGATGCCCTTTGGCTGC
7 JHB/1/66             99.0%  27.1%  AGGGTGGGTGAA--TGAAACCAGTCCTTTTACGGAGAAATACCTCCTTCCTCCCAAATTTGGAAGATGCCCTTTGGCTGC
  consensus/100%                     ...GTGGssGuA..sGAAAC..............AuuAA...................TTuGAAuATG..ssTsuGsTGC
  consensus/90%                      ...GTGGssGuA..sGAAAC..............AuuAA...................TTuGAAuATG..ssTsuGsTGC
  consensus/80%                      ...GTGGssGuA..TGAAAC..............AuuAA...................TTuGAAuATG..ssTsuGsTGC
  consensus/70%                      AGGGTGGGTGAA..TGAAACCAGsCCTTTTuCGGAGGAATACCTCCTsCCTCCCAAATTTGGAAGATGCCCsTTGGCTGC
```

```
                         cov     pid 1281        .         3         .         .         .         .         :         . 1360
1 Mississippi/80      100.0% 100.0%  ATTAAAAG----------------------CGGATTCGTTATGGCTACCGATGAAATCTCTCTCACTAT------ACTCC
2 TAYLOR/1233/47      100.0%  98.7%  ATTAAAAG----------------------CGGATTCGTTATGGCTACCGATGAAATCTCTCTCACTAT------ACTCC
3 Yamagata/26/81       99.0%  27.5%  AAAGGAAGAATCCATTCCAAAAATCCCAGATGGACTTCTAATTCCCACCAGTGGAACTGATACCACTGTAACTAAACCTA
4 Sao-Paulo/378/82     99.0%  28.3%  AAAGGAAGAATCTATTCCAAAAATCCCAGATGGCCTTCTAATCCCCACTAGTGGAACTGATACCACTGTAACCAAACCCA
5 Arbor/1/50           99.0%  27.1%  AAAGGAAGAATCCATTCCAAAAATCCCAGATGGCCTTCTAATTCCCACCAGTGGAACTGATACCACTGTAACCAAACCTA
6 Aichi/1/81           99.0%  28.0%  AAAGGAAGAATCCATTCCAAAAATCCCAGATGGCCTTCTAATTCCCACCAGTGGAACCGATACCACTGTAACCAAACCTA
7 JHB/1/66             99.0%  27.1%  AAAGGAAGAATCCATTCCAAAAATCCCAGATGGCCTTCTAATTCCCACCAGTGGAACCGATACCACTGTAACCAAACCTA
  consensus/100%                     AssuuAAG......................sGGssTssTsATssCsACsuuTGuAAssssTssCACTuT......ACsss
  consensus/90%                      AssuuAAG......................sGGssTssTsATssCsACsuuTGuAAssssTssCACTuT......ACsss
  consensus/80%                      AssuuAAG......................sGGssTssTsATssCsACCuuTGuAAssssTssCACTuT......ACsss
  consensus/70%                      AAAGGAAGAATCsATTCCAAAAATCCCAGATGGsCTTCTAATsCCCACCAGTGGAACsGATACCACTGTAACsAAACCsA
```

```
                         cov     pid 1361        .         .         .         4         .         .         .         . 1440
1 Mississippi/80      100.0% 100.0%  AAAGTATCAAATCAGGAGCCCA--------------GCTCGATCCCTATTG--------------GGAAATG--------
2 TAYLOR/1233/47      100.0%  98.7%  AAAGTATCAAATCAGGAGCCCA--------------GCTCGATCCCTATTG--------------GGAAATG--------
3 Yamagata/26/81       99.0%  27.5%  AAAGCAGAATTTTTGGAATCGATGACCTTATTATTGGTCTACTCTTTGTTGCAATCGTTGAAGCAGGAATTGGAGGCTAT
4 Sao-Paulo/378/82     99.0%  28.3%  AAAGCAGAATTTTTGGAATCGATGACCTTATTATTGGTTTGCTCTTTGTTGCAATCGTTGAAGCAGGAATTGGAGGCTAT
5 Arbor/1/50           99.0%  27.1%  AGAGCAGAATTTTTGGAATCGATGACCTCATTATTGGTTTGCTCTTTGTTGCAATCGTTGAAGCAGGAATTGGAGGCTAT
6 Aichi/1/81           99.0%  28.0%  AGAGCAGAATTTTTGGAATCGATGACCTCATTATTGGTTTGCTCTTTGTTGCAATCGTTGAAGCAGGAATTGGAGGCTAT
7 JHB/1/66             99.0%  27.1%  AGAGCAGAATTTTTGGAATCGATGACCTCATTATTGGTTTGCTCTTTGTTGCAATCGTTGAGGCAGGAATTGGAGGCTAT
  consensus/100%                     AuAGsAssAssTssGGAusCsA..............GsssusTCssTuTTG..............GGAAsTG........
  consensus/90%                      AuAGsAssAssTssGGAusCsA..............GsssusTCssTuTTG..............GGAAsTG........
  consensus/80%                      AuAGsAssAssTssGGAusCsA..............GsTsGsTCssTuTTG..............GGAAsTG........
  consensus/70%                      AuAGCAGAATTTTTGGAATCGATGACCTsATTATTGGTTTGCTCTTTGTTGCAATCGTTGAuGCAGGAATTGGAGGCTAT
```

```
                         cov     pid 1441        :         .         .         .         .         5         .         . 1520
1 Mississippi/80      100.0% 100.0%  ----------------------------------------AAACACCAGATA---------TTGACAAGACTGAAGCTTA
2 TAYLOR/1233/47      100.0%  98.7%  ----------------------------------------AAACACCAGATA---------TTGACAAGACTGAAGCTTA
3 Yamagata/26/81       99.0%  27.5%  CTGCTTGGAAGTAGAAAAGAATCAGGAGGAGGTGTGACAAAAGAATCAGCTGAAAAAGGGTTTGAAAAAATTGGAAATGA
4 Sao-Paulo/378/82     99.0%  28.3%  CTTCTTGGAAGTAGAAAAGAATCAGGAGGAGGTGTGACAAAAGAATCAGCTGAAAAAGGGTTTGAGAAAATTGGAAATGA
5 Arbor/1/50           99.0%  27.1%  CTGCTTGGAAGTAGAAAAGTATCAGGAGGAGGTGTGACAAAAGAATCAGCTGAAAAAGGGTTTGAGAAAATTGGAAATGA
6 Aichi/1/81           99.0%  28.0%  CTGCTCGGGAGTAGAAAAGAATCAGGAGGAGGTGTGACAAAAGAATCAGCTGAAAAAGGGTTCGAAAAAATTGGAAATGA
7 JHB/1/66             99.0%  27.1%  CTGCTTGGAAGTAGAAAAGAATCAGGAGGAGGTGTGACAAAAGAATCAGCTGAAAAAGGGTTTGAGAAAATTGGAAATGA
  consensus/100%                     ........................................AAusAsCAGsTu.........TsGAsAAuAsTGuAusTsA
  consensus/90%                      ........................................AAusAsCAGsTu.........TsGAsAAuAsTGuAusTsA
  consensus/80%                      ........................................AAusAsCAGsTu.........TTGAsAAuAsTGuAusTsA
  consensus/70%                      CTsCTsGGuAGTAGAAAAGsATCAGGAGGAGGTGTGACAAAAGAATCAGCTGAAAAAGGGTTTGAuAAAATTGGAAATGA
```

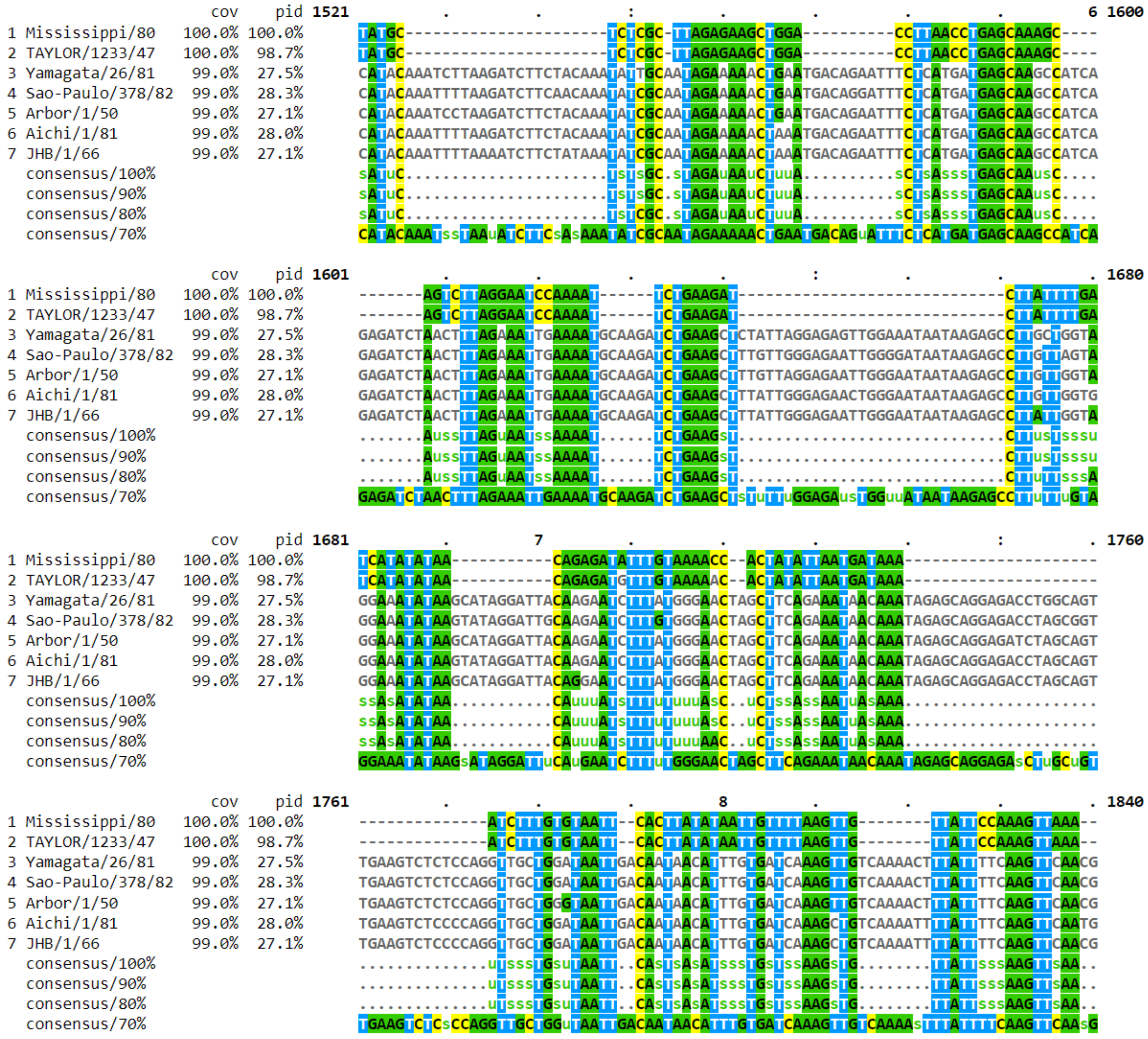

Figure S4. Multiple alignment of seven different strains of influenza c virus

Table S1. Docking energy of best complexes

Solution No	Score	Area (A^2)	ACE	Transformation
1	19704	3196	-2078.92	-2.07 0.75 0.87 -9.93 10.17 33.17
2	18860	3233.8	-2250.65	-1.32 0.88 0.12 -3.17 13.28 47.50

3	18434	3475.6	-2844.42	-1.96 -0.32 -2.07 40.40 -24.10 31.71
4	18194	3375.8	-2611.25	-1.34 0.48 0.27 2.41 12.31 54.99
5	18016	3118.4	-2074.01	-2.52 0.52 2.13 -7.28 -24.13 22.09
6	18008	2985.8	-2037.09	-2.16 0.47 1.19 -13.29 0.19 32.14
7	17940	3200.9	-2217.68	0.63 0.37 -2.96 7.62 25.25 31.62
8	17596	3588.8	-2639.53	-2.10 -0.79 1.59 -31.36 -10.55 14.90
9	17582	2892.2	-1638.76	-0.93 0.89 2.76 37.21 -40.46 53.94
10	17556	3015.7	-2034.68	-1.53 -0.38 -2.83 11.93 -36.30 41.55
